# QSAR and Pharmacophore Modeling of Nitrogen Heterocycles as Potent Human *N*-Myristoyltransferase (Hs-NMT) Inhibitors

**DOI:** 10.3390/molecules26071834

**Published:** 2021-03-24

**Authors:** Magdi E. A. Zaki, Sami A. Al-Hussain, Vijay H. Masand, Siddhartha Akasapu, Israa Lewaa

**Affiliations:** 1 Department of Chemistry, Faculty of Science, Imam Mohammad Ibn Saud Islamic University, Riyadh 13318, Saudi Arabia; sahussain@imamu.edu.sa; 2 Department of Chemistry, Vidya Bharati Mahavidyalaya, Amravati 444 602, Maharashtra, India; 3 Corden Pharma, Boulder, CO 80301, USA; asidhu09@gmail.com; 4 Department of Business Administration, Faculty of Business Administration, Economics and Political Science, British University in Egypt, Cairo 11837, Egypt; Israa.lewaa@bue.edu.eg

**Keywords:** human *N*-Myristoyltransferase, nitrogen heterocycles, QSAR, statistical analysis, pharmacophore modeling

## Abstract

*N*-myristoyltransferase (NMT) is an important eukaryotic monomeric enzyme which has emerged as an attractive target for developing a drug for cancer, leishmaniasis, ischemia-reperfusion injury, malaria, inflammation, etc. In the present work, statistically robust machine leaning models (QSAR (Quantitative Structure–Activity Relationship) approach) for Human NMT (Hs-NMT) inhibitory has been performed for a dataset of 309 Nitrogen heterocycles screened for NMT inhibitory activity. Hundreds of QSAR models were derived. Of these, the model 1 and 2 were chosen as they not only fulfil the recommended values for a good number of validation parameters (e.g., R^2^ = 0.77–0.79, Q^2^_LMO_ = 0.75–0.76, CCC_ex_ = 0.86–0.87, Q^2^-F^3^ = 0.74–0.76, etc.) but also provide useful insights into the structural features that sway the Hs-NMT inhibitory activity of Nitrogen heterocycles. That is, they have an acceptable equipoise of descriptive and predictive qualities as per Organisation for Economic Co-operation and Development (OECD) guidelines. The developed QSAR models identified a good number of molecular descriptors like solvent accessible surface area of all atoms having specific partial charge, absolute surface area of Carbon atoms, etc. as important features to be considered in future optimizations. In addition, pharmacophore modeling has been performed to get additional insight into the pharmacophoric features, which provided additional results.

## 1. Introduction

*N-*myristoyltransferase (NMT) or glycylpeptide *N-*tetradecanoyltransferase is an important cellular monomeric enzyme with a crucial role in the growth of various organisms and cellular proliferation [1,2,3,4,5]. It is ubiquitously distributed, which follows a sequentially ordered bi-bi mechanism to accomplish protein *N-*myristoylation [2,4,5]. The process of protein *N-*myristoylation is a two-step process which involves catalytic irreversible shifting of the myristoyl group to the *N-*terminal glycine residue of a number of substrate proteins [1,2,4,6,7,8]. It is a co- and post-translational modification resulting in increased protein lipophilicity, facilitates membrane localization, and optimizes binding with cellular membranes proteins and several lipophilic proteins [4,5]. It has been established that in cancerous states, the expression of NMT activity increases significantly in human adenocarcinoma, gallbladder cancer, and different types of cancers [1,2,3,4,5,6]. Similarly, the proliferative capacity is found to be linked with NMT activity in mammary epithelial cells, thereby making it a suitable target for cancer therapy. In humans and other higher eukaryotes, it exists in two predominant isoforms: NMT-1 (four distinct isoforms ranging from 49kDa to 68 kDa) and NMT-2 (single 65 kDa protein), with a mutual sequence similarity of ~77% [1,2,3,4,5,6]. Both the NMTs possess two distinct pockets, N and C terminus, for substrate binding. However, despite a high degree of sequence identity, the two NMTs have different substrate specificities for the peptide binding pocket present nearer to the C-terminus [5]. These two NMTs play a crucial role in apoptosis, signal transduction, cellular transformation, and oncogenesis. Due to their essential roles, NMT-1 and 2 have been recognized as plausible targets for the treatment of various diseases like leishmaniasis, ischemia-reperfusion injury, malaria, inflammation, Chagas disease, fungal infections, etc. [1,2,3,4,5,6].

Recently, Gilbert et al. [6,7,8,9,10] performed high-throughput screening (HTS) against the enzyme NMT followed by the synthesis and biological screening of a good number of Nitrogen heterocycles for NMT inhibitory activity. The Nitrogen heterocycles revealed moderate to high activity. Even though extensive SAR (Structure–Activity Relationship) analysis has been reported, simple visual inspection cannot identify concealed pharmacophoric patterns. In addition, while optimizing the ADMET profile (Absorption, Distribution, Metabolism, Excretion, and Toxicity), it is essential to retain the high activity. In such a situation, an in-depth understanding of obviously visible and hidden pharmacophoric features could be highly beneficial. Therefore, there is a need to carry advanced analysis using modern methods like Quantitative Structure–Activity Relationship (QSAR), pharmacophore modeling, etc. to identify additional and hidden features.

QSAR (Quantitative Structure–Activity Relationship) analysis involves an amalgamation of different fields (Chemistry, Statistics, Computer Science, etc.) to identify salient and concealed pharmacophoric patterns [11,12]. A successful QSAR analysis provides in-depth understanding of important structural features as well as helps to predict the desired bio-activity before the actual synthesis and bio-screening of a congeneric molecule [13,14,15,16]. Therefore, researchers use machine-learning-based QSAR approach for a dataset of congeneric molecules to get ideas about prominent features, which are then further used for optimization of a selected lead/drug candidate. Therefore, a dataset of structurally diverse Nitrogen heterocycles has been used in the present work to develop balanced QSAR models. The newly derived QSAR models comprise interpretable molecular descriptors in terms of structural features that have good correlation with Human *N-*myristoyltransferase-1 (Hs-NMT-1) inhibitory activity of Nitrogen heterocycles. A rational and conceivable discernment of structural features deciding the Hs-NMT inhibitory activity of Nitrogen heterocycles would be of prodigious significance to future drug discovery efforts.

Another thriving Computer Aided Drug Designing CADD approach is pharmacophore modeling, which is routinely performed to identify important structural features. In the present work, QSAR and pharmacophore modeling have been concurrently performed to identify the consensus and complementary structural features which govern the anti-Hs-NMT-1 activity of Nitrogen heterocycles.

## 2. Experimental Methodology

A QSAR analysis based on appropriate selection of dataset, molecular descriptors, feature selection algorithm, validation method, and correlation in terms of structural features (i.e., OECD guidelines) is useful to derive a QSAR model having a good balance of external predictive ability (Quantitative/Predictive QSAR) and interpretation of model/molecular descriptors in terms of structural features (Qualitative/Descriptive QSAR) [13,14,15,16,17,18,19,20]. Therefore, the standard procedure has been followed to derive a balanced QSAR model for inhibitory activity of Nitrogen heterocycles for Human NMT. More details about the procedure followed in the present work are available in the literature [11,17,21,22,23,24,25].

### 2.1. Selection of Dataset

**Figure 1:**
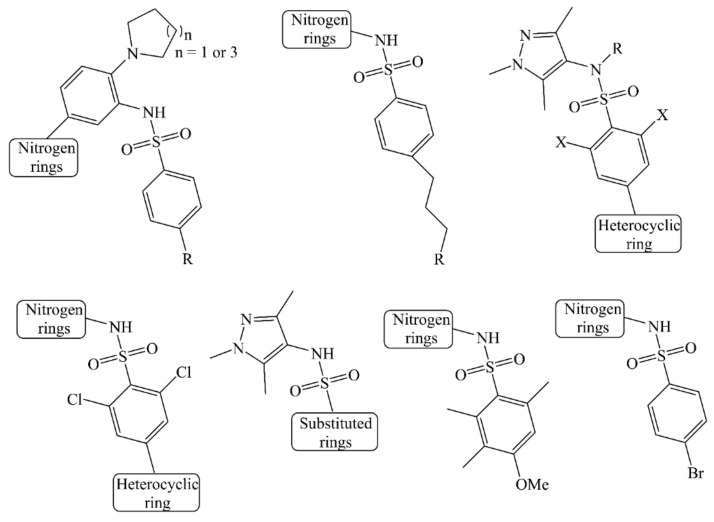
Structural variations in Nitrogen heterocycles used for Quantitative Structure–Activity Relationship (QSAR) analysis.

A dataset of structurally diverse 309 Nitrogen heterocycles, which have been tested for their activity for Human NMT-1, has been selected for QSAR analysis in the present work [6,7,8,9,10]. The dataset compromises a variety of molecules with varying substituents like positional and ring isomers, heterocyclic analogues, etc. Thus, covering a broad chemical space required for deriving a robust QSAR model. The reported IC_50_ values range from 0.002 uM to 107,000 uM, which were converted to pIC_50_ (−log_10_IC_50_) before deriving the QSAR model. The SMILES (Simplified Molecular-Input Line-Entry System) notations, IC_50_, and pIC_50_ for all the Nitrogen heterocycles are present in supplementary material (see Table S1). Herein, in Table 1, we have presented five most and least active molecules as examples only. In addition, the common scaffolds have been presented in Figure 1.

The 2D-structures of all the Nitrogen heterocycles were drawn using ACD ChemSketch Freeware (www.acdlabs.com (accessed on 23 February 2021)). Thereafter, conversion to 3D-using Avogadro ver. 1.02 (https://avogadro.cc/ (accessed on 23 February 2021)) using MMFF94 force field for geometry optimization and partial charge assignment was performed. The following parameters were used for geometry optimization: ForceField: MMFF94, Algorithm: Steepest Descent, number of steps used for optimization: 1000.

### 2.2. Calculation and Pruning of Molecular Descriptors

PyDescriptor [26] and PaDEL [27] were used for molecular descriptor calculation using 3D-optimized structures of Nitrogen heterocycles. While calculating solvent-accessible surface area using PyDescriptor, the dot density was set to 4 in PyMOL to get a higher accuracy. This resulted in generation of more than 29,000 molecular descriptors for each molecule. In the next step, objective feature selection (OFS) was performed to reduce the pool of molecular descriptors. OFS involved the removal of constant, nearly constant (95% molecules), and highly correlated molecular descriptors (|R| > 0.90) [17,23,24,25]. OFS resulted in a reduced cluster of 596 molecular descriptors, which still covers a broad descriptor space due to the presence of 1D- to 3D-molecular descriptors. This condensed pool was used for subjective feature selection (SFS).

### 2.3. Subjective Feature Selection (Model Building)

**Figure 2:**
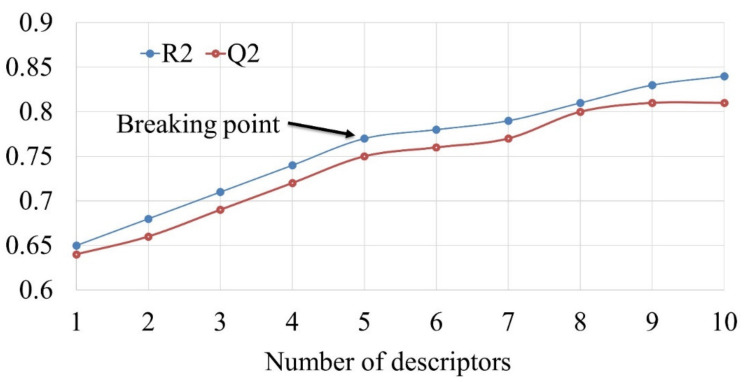
Plot of the number of descriptors against Coefficient of Determination R2 and Leave-One-Out Coefficient of Determination Q2 to identify the optimum number of descriptors.

The process of SFS was accomplished using QSARINS-2.2.4 [28,29] with default settings, except the number of generations was set to 10,000. The Genetic Algorithm (GA) module of QSARINS-2.2.4 uses Q2 as a fitting parameter to avoid over fitting and inclusion of redundant variables during model building [28,29]. A crucial decision in developing a successful QSAR model is when to stop adding molecular descriptors to the model. In the present work, a graph was drawn between the number of descriptors involved in the models and Q2 value to obtain the so-called breaking point. Consequently, the number of molecular descriptors corresponding to the breaking point was considered optimum for model building. The graph between the numbers of variables used in the models against Q2 value is depicted in Figure 2. From the Figure 2, it is clear that the breaking point corresponds to five variables, that is, optimum number of descriptors to be used is five. Therefore, QSAR models with more than five descriptors were rejected.

**Figure 3:**
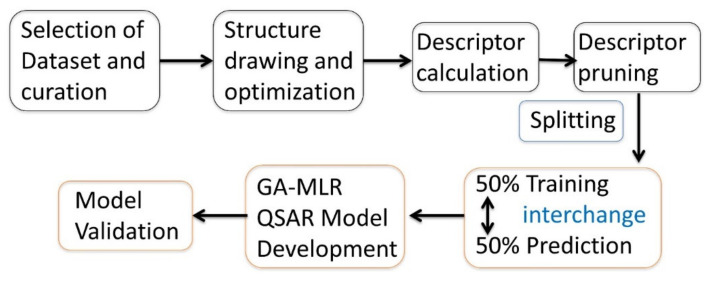
Procedure adopted in the current work to develop acceptable QSAR models.

A major reason to derive any statistically acceptable genetic algorithm–multiple linear regression (GA-MLR)-based QSAR model is to gain thorough information about the structural features that decide the desired activity (Descriptive QSAR) [13,14]. To achieve this objective, the strategy [11,30] involves splitting of dataset into two equal sets: 50% training set and 50% prediction set. The training set was used to find the most relevant and appropriate number of molecular descriptors for QSAR model, whereas the prediction set was used for external validation, i.e., to check the external prediction ability (Predictive QSAR). Then, the training and the prediction sets were exchanged for descriptor selection, model building, and proper validation, thereby ensuring the identification of relevant molecular descriptors, in turn, the structural features that influence the HsNMT-1 activity of Nitrogen heterocycles. The strategy has been depicted in Figure 3.

### 2.4. Validation of QSAR Models

After building a QSAR model, it is an essential step to validate its performance, that is, to judge its external predictive ability and statistical robustness for its further usage in drug/lead optimizations [12,13,14,15,18,20,21,31,32,33,34,35]. Therefore, not only thorough internal validations (cross-validation approach) and Y-scrambling were performed, but an external prediction set of 50% molecules was also used to validate the statistical performance of the model. In addition, Williams plot was used to assess the applicability domain of the developed QSAR model. To add further, the following rules were used to select and validate a model [11,17,22,23,24]: R2tr ≥ 0.6, Q2loo ≥ 0.5, Q2LMO ≥ 0.6, R2 > Q2, R2ex ≥ 0.6, RMSEtr < RMSEcv, ΔK ≥ 0.05, CCC ≥ 0.80, Q2-Fn ≥ 0.60, r2m ≥ 0.5, (1-r2/ro2) < 0.1, 0.9 ≤ k ≤ 1.1 or (1-r2/r’o2) < 0.1, 0.9 ≤ k’ ≤ 1.1,| ro2− r’o2| < 0.3 with RMSE and MAE as low as possible. The Q2LMO value reported herein is mean value of 2000 repetitions with 30% of the objects randomly excluded from the training set each time. The external predictive ability of model was assessed using external validation parameters, viz., RMSEex, MAEex, R2ex, Q2F1, Q2F2, Q2F3, and CCCex. All QSAR models that do not achieve the recommended lower-limit values for above statistical parameters were directly rejected.

### 2.5. Pharmacophore Modeling

To get a consensus pharmacophore model, the molecules in the data set were aligned using the lowest energy conformer of the most active compound. Then, LIQUID 1.0, a free PyMOL [36] plugin, was used to generate consensus pharmacophore model using default settings. Whereas, for pharmacophore modeling for x-ray resolved crystal structures of reported inhibitors, the pdb files (6FZ5 and 3IWE) were downloaded from a publicly available database (www.rcsb.org (accessed on 23 February 2021)). After that, the crystallized ligands were extracted without any modification or optimization, that is, the x-ray resolved crystal structure of extracted ligand was used as such to generate the pharmacophore model using LIQUID 1.0.

## 3. Results and Discussions

In the present work, the QSAR analysis was aimed to recognize important structural features responsible for HsNMT-1 inhibitory activity of Nitrogen heterocycles. The developed five parametric GA-MLR-based QSAR models have acceptable statistical performance with the presence of easily understandable molecular descriptors, thereby leading to a relevant explanation and association with structural features. Even though, in the present analysis, a straight evaluation of IC50 values of the molecules of the dataset has been performed to explain the effect of a specific descriptor, it is important to note that the synergic or inverse effect of unknown factors or other molecular descriptors could have a substantial influence in deciding the final IC50 value of a molecule. It is essential to understand that no single molecular descriptor is able to completely explain the observed distribution of IC50 for such a diverse set of molecules. That is, the performance of the developed QSAR model relies on the concomitant use of constituent molecular descriptors. The derived five-parametric GA-MLR QSAR models are as follows:

***Model-1:*** pIC50 = 0.928 (± 0.703) + 0.028 (± 0.008) * C_AbSA + 0.009 (± 0.001) * all_HASA2-0.142 (± 0.069) * fNH4B + 0.554 (± 0.311) * fringNH2A -0.241 (± 0.133) * flipoH3B

R2tr = 0.788, R2adj. = 0.781, R2tr-R2adj. = 0.007, LOF = 0.831, Kxx = 0.296, ΔK = 0.063, RMSEtr = 0.853, MAEtr = 0.699, RSStr = 112.659, CCCtr = 0.882, s = 0.87, F = 110.953, R2cv (Q2loo) = 0.77, R2-R2cv = 0.019, RMSEcv = 0.89, MAEcv = 0.729, PRESScv = 122.628, CCCcv = 0.871, Q2LMO = 0.764, R2Yscr = 0.033, Q2Yscr = -0.047, RMSEex = 0.907, MAEex = 0.743, PRESSext = 126.77, R2ex = 0.757, Q2-F1 = 0.755, Q2-F2 = 0.755, Q2-F3 = 0.76, CCCex = 0.867, R2-ExPy = 0.757, R’o2 = 0.714, k’ = 0.973, 1-(R2/ R’o2) = 0.057, r’2m = 0.6, Ro2 = 0.755, k = 0.998, 1-(R2-ExPy/ Ro2) = 0.003, r2m = 0.723

***Model-2:*** pIC50 = 1.574 (± 0.618) + 0.026 (± 0.008) * C_AbSA + 0.008 (± 0.001) * all_HASA2 -0.19 (± 0.068) * fNH4B + 0.772 (± 0.336) * fringNH2A -0.287 (± 0.139) * flipoH3B

R2tr = 0.771, R2adj. = 0.763, R2tr-R2adj. = 0.008, LOF = 0.882, Kxx = 0.258, ΔK = 0.081, RMSEtr = 0.878, MAEtr = 0.727, RSStr = 118.695, CCCtr = 0.87, s = 0.896, F = 99.365, R2cv (Q2loo) = 0.75, R2-R2cv = 0.021, RMSEcv = 0.917, MAEcv = 0.758, PRESScv = 129.517, CCCcv = 0.859, Q2LMO = 0.745, R2Yscr = 0.033, Q2Yscr = -0.048, RMSEex = 0.876, MAEex = 0.725, PRESSext = 118.967, R2ex = 0.777, Q2-F1 = 0.776, Q2-F2 = 0.776, Q2-F3 = 0.771, CCCex = 0.871, R2-ExPy = 0.777, R’o2 = 0.705, k’ = 0.974, 1-(R2/ R’o2) = 0.093, r’2m = 0.568, Ro2 = 0.776, k = 0.999, 1-(R2-ExPy/ Ro2) = 0.001, r2m = 0.758 

**Figure 4:**
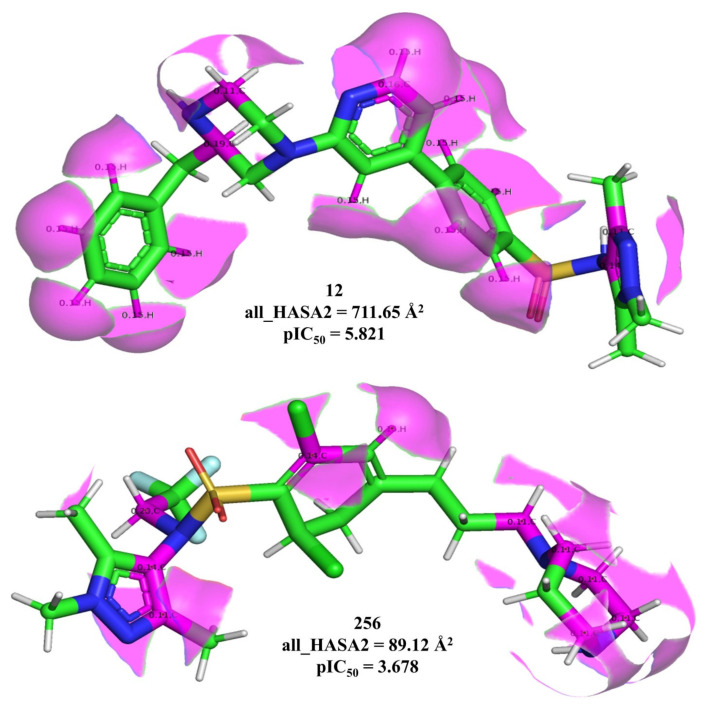
Representation of all_HASA2 using molecule 256 and 12 as examples only (all_HASA2 shown in magenta color).

The molecular descriptor all_HASA2 represents the solvent accessible surface area (Å2) of all atoms having a partial charge in the range +0.10 to +0.20, which in turn indicates the importance of hydrophobic atoms. all_HASA2 has a positive coefficient in both the developed QSAR models, therefore, increasing its value could lead to better activity for HsNMT-1. This molecular descriptor has been depicted in Figure 4 using molecule 256 (least all_HASA2) and 12 (highest all_HASA2) as examples only.

Brand et al. [10] highlighted the importance of reduction of polar surface area (PSA) to increase Blood-Brain Barrier (BBB) penetration ability of Nitrogen heterocycles. A decrease in PSA is possible only if the number of negatively charged Nitrogen and/or Oxygen atoms is minimized, which in turn could lead to an increase in the number of positively charged atoms. Interestingly, an increase in the number of positively charged atoms could lead to an increased value of all_HASA2. Therefore, it is rational to say that an increase in all_HASA2 could also lead to enhanced BBB penetration ability.

As the calculation of all_HASA2 involves solvent-accessible surface area, it is indirectly related to the size of a molecule. It is observed that molecules with higher activity for Hs-NMT-1 have a larger size and all_HASA2 compared to less active molecules (see Supplementary Material).

The molecular descriptor C_AbSA corresponds to absolute surface area due to Carbon atoms in the molecule, which in turn points out the importance of hydrophobic atoms. The absolute surface area is the difference between solvent-accessible surface area and molecular surface area (MSA). C_AbSA has positive coefficients in the developed QSAR models, which indicates that as the value of C_AbSA increases, the HsNMT inhibition ability also increases. In our previous work [11], we have reported that C_AbSA has a positive correlation with the Tb-NMT inhibitory activity of pyrazole Nitrogen heterocycles. This molecular descriptor also highlights the importance of the number of Carbon atoms in the molecule. Therefore, increasing the number of Carbon atoms is a good idea to have better inhibitory activity for NMT. 

The two molecular descriptors all_HASA2 and C_AbSA indicate that the presence of hydrophobic atoms is important for Hs-NMT inhibitory activity. This observation is also supported by the pharmacophore modeling and crystal structure of Hs-NMT-1 in complex with various ligands. Recently, Kersten et al. [37] reported that sulphonamide derivatives bearing Nitrogen heterocycles interact with hydrophobic residue Tyr296 (pdb code 3IWE and 6FZ5), which vindicates the importance of the presence of hydrophobic atoms in the ligand.

The molecular descriptor fringNH2A stands for the frequency of occurrence of H atoms exactly between 1 to 2 angstroms from the ring Nitrogen atoms. If the same Hydrogen atom was simultaneously present at zero to one angstrom from any other ring Nitrogen atom, then it was excluded during the calculation of fringNH2A. It has a positive coefficient in the developed models, therefore, the number of Hydrogen atoms in the vicinity of ring Nitrogen atoms is a favorable combination to be used for lead/drug optimization for HsNMT-1. Since Hydrogen is the smallest element, it implies that there should be minimum bulk in the vicinity of ring Nitrogen atoms. Therefore, in future modifications, steric bulk nearer to ring Nitrogen atoms should be avoided to have better activity against HsNMT-1. 

**Figure 5:**
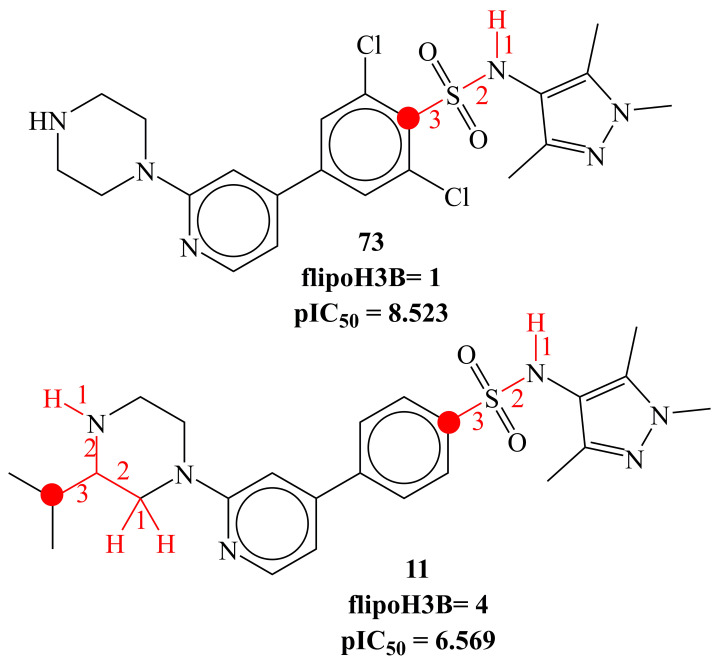
Representation of flipoH3B (red-colored Hydrogen atoms and bonds) using molecule 11 and 73 as examples only.

The molecular descriptor flipoH3B indicates the frequency of occurrence of Hydrogen atoms exactly at three bonds from lipophilic atoms (see Figure 5). If the same hydrogen atom is simultaneously present at one or two bonds from any other lipophilic atom, then it was excluded during the calculation of flipoH3B. This molecular descriptor has a negative coefficient in developed model 1, therefore, its value should be kept as low as possible. Again, due to the lowest size of Hydrogen, it points out that there should be more bulkiness at three bonds from lipophilic atoms to achieve better activity against HsNMT.

**Figure 6:**
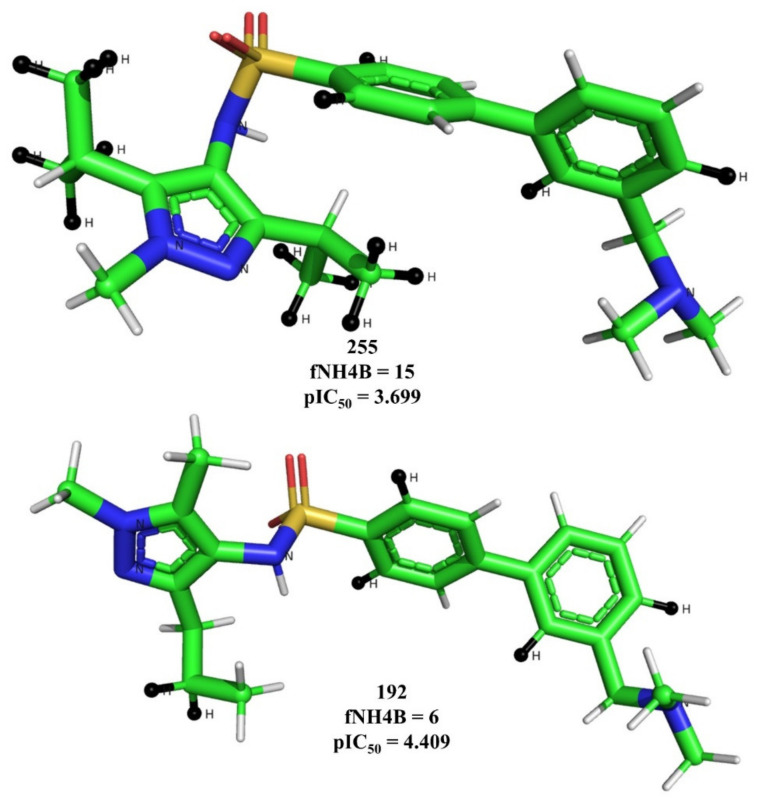
Depiction of fNH4B (black-colored Hydrogen atoms) using molecule 149 and 192 as representative examples only.

The molecular descriptor fNH4B represents the frequency of occurrence of Hydrogen atoms exactly at four bonds from Nitrogen atoms. If the same Hydrogen atom is also present at less than four bonds from any other Nitrogen atom, then it was excluded during the calculation of fNH4B. The negative coefficient for this molecular descriptor in model 1 and 2 indicates that increasing the number of such Hydrogen atoms could lead to lower activity against HsNMT-1. Figure 6 contains the pictorial representation of fNH4B using molecule 255 and 192 as examples.

**Figure 7:**
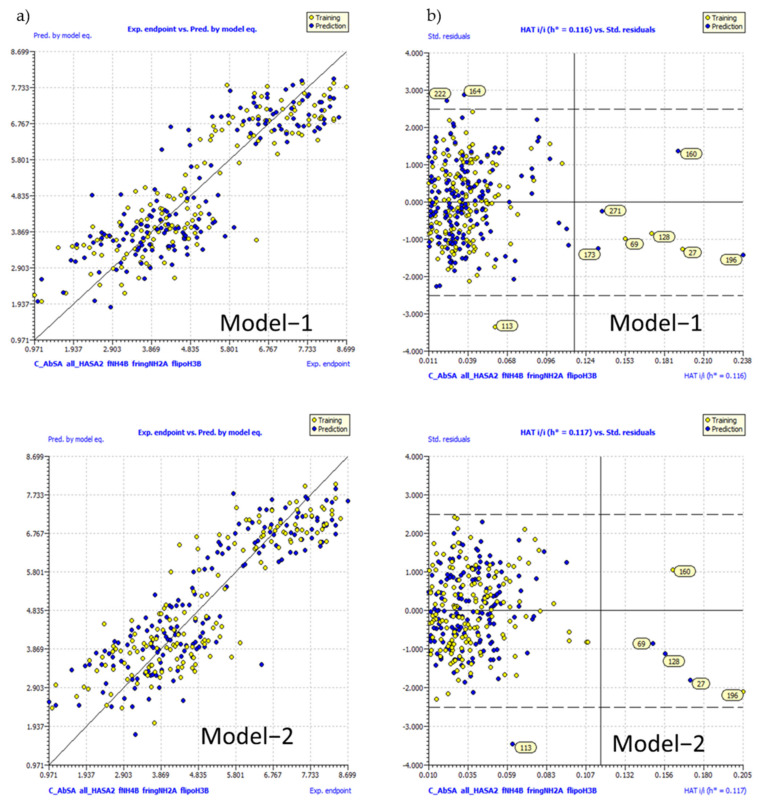
Different graphs associated with the derived QSAR models. (**a**) Experimental vs. predicted value graph, (**b**) Williams plot for model 1 and 2.

A simple comparison of the two models indicates that they contain identical molecular descriptors; therefore, the strategy is highly successful in identifying most relevant molecular descriptors. This also points out the statistical stability of the models. The developed QSAR models have successfully satiated values for many fitting, cross-validation, and external validation parameters. A high value of R2tr, R2cv (Q2loo), Q2LMO, and CCCcv indicates the statistical robustness of the developed models [16,20,32,34,37,38,39]. In addition, Y-scrambling results also confirm this observation. The external predictive ability of the developed models is confirmed on the basis of high value of Q2-Fn, CCCex, and R2ex [16,20,32,34,38,39,40]. For the developed QSAR models, R2adj. and R2 have negligible difference, which signifies that the models contain a suitable number of molecular descriptors. A high Fischer ratio (F) value for the developed models further pointed out the statistical significance of the developed models. The formulae to calculate the statistical parameters are available in supplementary material (see Tables S2 and S3, Figures S1 and S2). Various graphs associated with the models have been depicted in Figure 7.

From the graphs (see Figure 7), it is clear that the models are statistically acceptable and useful for the prediction of Hs-NMT inhibitory activity for future developments.

## 4. Pharmacophore Modeling

**Figure 8:**
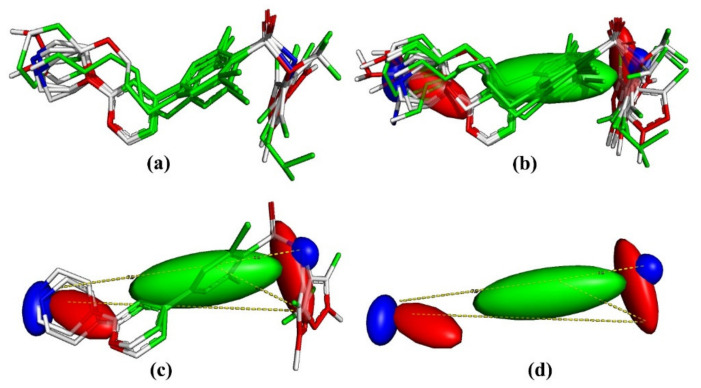
Consensus pharmacophore model, (**a**) aligned ligands, (**b**) pharmacophore model with molecules, (**c**) pharmacophore model with molecules and distances using representative examples, (**d**) different regions with distances only.

**Figure 9:**
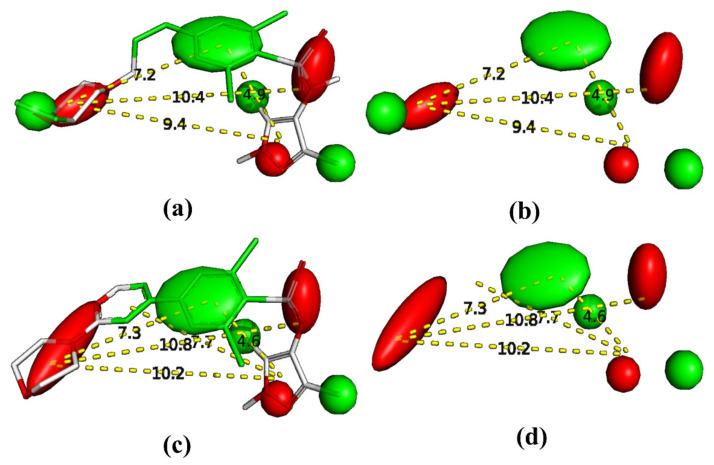
(**a**) Pharmacophore model with crystallized ligands-pdb 6FZ5. (**b**) Pharmacophore model without crystallized ligands-pdb 6FZ5. (**c**) Pharmacophore model with crystallized ligands-pdb 3IWE. (**d**) Pharmacophore model without crystallized ligands-pdb 3IWE.

Pharmacophore modeling is a well-established thriving branch of CADD that is performed to identify crucial structural features. The consensus pharmacophore model reveals that the pharmacophoric pattern (see Figure 8) consists of a larger hydrophobic region (green colored), two H-bond acceptors (red colored) at a distance of 11.2 Å from each other, and two H-bond donor regions at a distance of 4.0 Å and 7.9 Å from the center of the benzene ring attached to sulfnoamide group (green colored). The pharmacophore modeling highlighted the importance of the hydrophobic character of the central benzene ring and its vicinal atoms. The same observation is also supported by the presence of all_HASA2 and C_AbSA in the QSAR model as well as recent crystal structures for sulphonamide derivatives bearing Nitrogen heterocycles with Hs-NMT-1 (see Figure 9). A comparison of Figure 8 and Figure 9 indicates that the consensus pharmacophore model and the pharmacophore model obtained using the x-ray resolved crystal structure of extracted ligands have good similarity with each other, especially with respect to the presence of the hydrophobic region (green colored) at the center and two H-bond acceptors (red colored). Thus, QSAR and pharmacophore modeling led to identification of consensus and complementary structural features and vindicated by recent crystal structures [37].

## 5. Conclusions

In the present work, QSAR models having a good balance of external predictive ability and mechanistic interpretation were derived. The analysis pointed out that some structural features like solvent accessible surface area of all atoms having specific partial charge (range +0.1 to +0.2), absolute surface area of carbon atoms, and presence/absence of hydrogen from ring/non-ring Nitrogen atoms at specific distances as important structural features. The developed QSAR models have good external predictive ability, which could be used to identify novel hits, thus broadening their usefulness. In addition, QSAR and pharmacophore modeling concurrently identified consensus and complementary structural features.

## Figures and Tables

**Table 1:** Five most and least active Nitrogen heterocycles against Human *N-*myristoyltransferase (Hs-NMT) molecules (arranged according to IC_50_ values).

S.N.	SMILES	IC_50_ (µM)	pIC_50_ (M)
20	Cn(n1)c(C)c(c1C)N(C(F)F)S(=O)(=O)c(c(Cl)c2)c(Cl)cc2CCCO[C@H](C3)C[C@@H](N4C)CC[C@H]34	0.002	8.699
73	Cn(n1)c(C)c(c1C)NS(=O)(=O)c(c(Cl)c2)c(Cl)cc2-c3cc(ncc3)N4CCNCC4	0.003	8.523
2	Cn(n1)c(C)c(c1C)NS(=O)(=O)c(cc2)c(Cl)cc2-c3cc(ncc3)N4CCNCC4	0.004	8.398
78	CC(C)Cc1c(c(C)n(n1)C)NS(=O)(=O)c(c(Cl)c2)c(Cl)cc2-c3cc(ncc3)N4CCNCC4	0.004	8.398
95	C1CN(C)CCC1CCCCc2cc(Cl)c(c(Cl)c2)S(=O)(=O)Nc(c3C)c(C)n(n3)C	0.004	8.398
293	CC(C)Cc1ccc2ncccc2c1NS(=O)(=O)c1ccc(CCCO[C@H]2C[C@@H]3CC[C@H](C2)N3C)cc1	27000	1.569
82	Cc1c(C)c(OC)cc(C)c1S(=O)(=O)Nc(c2C)cccn2	70000	1.155
83	Cn1ncc(c1C)NS(=O)(=O)c2c(Cl)cc(Br)cc2Cl	70800	1.15
84	Cn(n1)c(C)c(c1C)NS(=O)(=O)c(c2F)ccc(Br)c2	87000	1.06
111	Cn(n1)c(C)c(c1C)NS(=O)(=O)c(c2C)ccc(Br)c2	107000	0.971

## Data Availability

Not Applicable.

## Supplementary Materials

The following are available online, Table S1: SMILES notations, IC50, pIC50, and molecular descriptors used in the QSAR models for all the sulphonamide derivatives used in the present, Table S2: Details regarding performance of model 1, Table S3: Details regarding performance of model 2, Figure S1: Different graphs associated with model 1 (a) graph of experimental vs. residual values (b) Y-scrambling plot, Figure S2: Different graphs associated with model 2 (a) graph of experimental vs. residual values (b) Y-scrambling plot, Statistical parameters for used for validation of QSAR models

## Sample Availability

Samples of the compounds are not available from the authors.

## Abbreviations

SMILES—Simplified Molecular-Input Line-Entry System; GA—Genetic Algorithm; MLR—Multiple Linear Regression; QSAR—Quantitative Structure−Activity Analysis; WHO—World Health Organization; ADMET—Absorption, Distribution, Metabolism, Excretion, and Toxicity; OLS—Ordinary Least Square; SARS-CoV—Severe Acute Respiratory Syndrome coronavirus; QSARINS-QSAR Insubria, OECD—Organisation for Economic Co-operation and Development/ S.N.-Serial Number.
