# Supplementary material for: QSAR and Pharmacophore Modeling of Nitrogen Heterocycles as Potent Human N-Myristoyltransferase (Hs-NMT) Inhibitors
Source: Molecules. 2021 Mar 24;26(7):1834. doi: 10.3390/molecules26071834 (PMC8038050; doi:10.3390/molecules26071834)
Supplement: Supplementary file 1 [file molecules-26-01834-s001.zip › Supplementary material for conversion molecules-1138940.pdf]

QSAR and Pharmacophore modeling of Nitrogen heterocycles as potent Human N-Myristoyltransferase (Hs-NMT) inhibitors

Magdi E.A. Zaki*[1], Sami A. Al-Hussain[1], Vijay H. Masand[2], Siddhartha Akasapu[2], Israa Lewaa[3]

[1] Department of Chemistry, Faculty of Science, Al-Imam Mohammad Ibn Saud Islamic University, Riyadh 13318, Saudi Arabia (Mezaki@imamu.edu.sa)

[2] Department of Chemistry, Vidya Bharati Mahavidyalaya, Amravati, Maharashtra, India- 444 602 (vijaymasand@gmail.com)

[3] Corden Pharma, Colorado, USA (asidhu09@gmail.com)

[4] Assistant Lecturer of Statistics, Department of Business Administration, Economics and Political Science, British University in Egypt, Cairo, Egypt (Israa.lewaa@bue.edu.eg)

Table S1: SMILES notations, IC_{50}, pIC_{50} and molecular descriptors used in the QSAR models for all the Sulphonamide derivatives used in the present work

SN	SMILES	IC_{50} HsNMT(μM)	pIC_{50}HsNMT (M)	C_AbSA	all_HASA2	fNH4B	fringNH2A	flipoH3B	fSC5B	fNC6B
1	C1CNCCN1CCCc2ccc(cc2)S(=O)(=O)Nc(c3C)c(C)n(n3)C	1000	3	53.97946	171.3806	4	1	1	2	0
2	Cn(n1)c(C)c(c1C)NS(=O)(=O)c(cc2)c(Cl)cc2-c3cc(ncc3)N4CCNCC4	0.004	8.398	31.30345	575.3866	1	1	1	2	0
3	Cn(n1)c(C)c(c1C)NS(=O)(=O)c(cc2)ccc2-c3cc(ncc3)N4CCNCC4	0.008	8.097	29.4418	610.7625	2	1	1	2	0
4	Cn(n1)c(C)c(c1C)NS(=O)(=O)c(c(F)c2)cc(F)c2-c3cc(ncc3)N4CCNCC4	0.012	7.921	53.22856	516.5901	1	1	1	2	0
5	Cn(n1)c(C)c(c1C)NS(=O)(=O)c(c2C)ccc(c2)-c3cc(ncc3)N4CCNCC4	0.007	8.155	53.24039	583.4348	1	1	1	2	0
6	Cn(n1)c(C)c(c1C)NS(=O)(=O)c(cc2)c(F)cc2-c3cc(ncc3)N4CCNCC4	0.007	8.155	29.83902	585.6846	1	1	1	2	0
7	Cn(n1)c(C)c(c1C)NS(=O)(=O)c(c(F)c2)c(F)cc2-	0.037	7.432	50.8	524.93	0	1	1	2	0

	c3cc(ncc3)N4CCNCC4			5343	9					
8	Cn(n1)c(C)c(c1C)NS(=O)(=O)c(cc2)ccc2-c3cc(ncc3)N(C[C@H]4C)CCN4	0.01	8	49.06482	597.6872	2	1	4	2	0
9	Cn(n1)c(C)c(c1C)NS(=O)(=O)c(cc2)ccc2-c3cc(ncc3)N(CCN4)C[C@H]4c5ccccc5	2.46	5.609	42.58752	687.067	4	1	4	2	0
10	Cn(n1)c(C)c(c1C)NS(=O)(=O)c(cc2)ccc2-c3cc(ncc3)N(C[C@H]4C)C[C@@H](C)N4	0.061	7.215	56.59404	615.2313	2	1	6	2	0
11	CC(C)[C@@H]1CN(CCN1)c(ncc2)cc2-c3ccc(cc3)S(=O)(=O)Nc(c4C)c(C)n(n4)C	0.27	6.569	53.47652	648.6231	8	1	4	2	0
12	c1ccccc1C[C@@H]2CN(CCN2)c(ncc3)cc3-c4ccc(cc4)S(=O)(=O)Nc(c5C)c(C)n(n5)C	1.51	5.821	49.58124	711.6528	2	1	4	2	1
13	Cn(n1)c(C)c(c1C)N(C(F)F)S(=O)(=O)c(c(Cl)c2)c(Cl)cc2CCCN3CCCN(C)CC3	0.007	8.155	67.14822	541.6297	2	0	0	2	0
14	Cn(n1)c(C)c(c1C)N(C(F)F)S(=O)(=O)c(c(Cl)c2)c(Cl)cc2CCCN3CCCNCC3	0.008	8.097	70.58742	519.3502	2	1	1	2	0
15	Cn(n1)c(C)c(c1C)N(C(F)F)S(=O)(=O)c(c(Cl)c2)c(Cl)cc2CCCCCCn3ccnc3	0.14	6.854	55.35437	515.534	2	0	0	2	1
16	Cn(n1)c(C)c(c1C)N(C(F)F)S(=O)(=O)c(c(Cl)c2)c(Cl)cc2CCCCCCn3ccnc3C	0.059	7.229	58.99508	544.3936	2	0	0	2	1
17	Cn(n1)c(C)c(c1C)N(C(F)F)S(=O)(=O)c(c(Cl)c2)c(Cl)cc2CCCCCCn3ccnc3CC	0.42	6.377	66.47487	569.0989	5	0	0	2	1
18	CC(C)c1nccn1CCCCCCc2cc(Cl)c(c(Cl)c2)S(=O)(=O)N(C(F)F)c(c3C)c(C)n(n3)C	0.5	6.301	70.26071	605.2743	8	0	0	2	1
19	Cn(n1)c(C)c(c1C)N(C(F)F)S(=O)(=O)c(c(Cl)c2)c(Cl)cc2CCCO[C@@H](C3)C[C@@H](N4C)CC[C@H]34	0.062	7.208	64.93218	597.9512	1	0	0	2	2
20	Cn(n1)c(C)c(c1C)N(C(F)F)S(=O)(=O)c(c(Cl)c2)c(Cl)cc2CCCO[C@H](C3)C[C@@H](N4C)CC[C@H]34	0.002	8.699	66.34833	601.5057	1	0	0	2	2
21	Cn(n1)c(C)c(c1C)N(C(F)F)S(=O)(=O)c(c(Cl)c2)c(Cl)cc2CCCO[C@H](C34)CN(CC3)CC4	0.017	7.77	62.14368	568.1349	1	0	2	2	1
22	Cn(n1)c(C)c(c1C)N(C(F)F)S(=O)(=O)c(c(Cl)c2)c(Cl)cc2-c3cc(ncc3)N4CCCNC4	0.017	7.77	46.54691	525.9326	0	1	1	2	0
23	Cn(n1)c(C)c(c1C)N(C(F)F)S(=O)(=O)c(c(Cl)c2)c(Cl)cc2CCCO[C@H](C3(C)C)CC(C)(C)N(C)C3	1.7	5.77	63.40854	621.6712	7	0	0	2	2
24	Cn(n1)c(C)c(c1C)N(C(F)F)S(=O)(=O)c(c(Cl)c2)c(Cl)cc2CCCO[C@@H](CC3)CC[C@@H]3N(C)C	0.005	8.301	66.46057	614.3663	4	0	0	2	2

25	Cn(n1)c(C)c(c1C)N(C(F)F)S(=O)(=O)c2c(Cl)cc(cc2Cl)[C@H]3CC[C@H](CC3)N(C)C	0.12	6.921	56.92558	505.0405	4	0	0	2	0
26	Cn(n1)c(C)c(c1C)N(C(F)F)S(=O)(=O)c2c(Cl)cc(cc2Cl)[C@H]3CC[C@@H](CC3)N(C)C	0.35	6.456	56.18557	506.844	4	0	0	2	0
27	Cn(n1)c(C)c(c1C)N(C(F)F)S(=O)(=O)c(c(Cl)c2)c(Cl)cc2CCCC[C@@H]3CN(C)CCO3	0.043	7.367	59.04043	554.1346	2	0	2	2	1
28	Cn(n1)c(C)c(c1C)N(C(F)F)S(=O)(=O)c(c(Cl)c2)c(Cl)cc2CCCCCCN(CC)CC	0.042	7.377	67.33201	620.7645	2	0	0	2	1
29	CC(C)CN(C)CCCCCCc1cc(Cl)c(c(Cl)c1)S(=O)(=O)N(C(F)F)c(c2C)c(C)n(n2)C	0.33	6.481	71.50744	640.3132	8	0	0	2	1
30	Cn(n1)c(C)c(c1C)N(C(F)F)S(=O)(=O)c(c(Cl)c2)c(Cl)cc2CCCCCCN(C)CC	0.024	7.62	61.23441	594.4399	2	0	0	2	1
31	CC(C)N(C)CCCCCCc1cc(Cl)c(c(Cl)c1)S(=O)(=O)N(C(F)F)c(c2C)c(C)n(n2)C	0.18	6.745	62.10321	611.3011	2	0	0	2	1
32	Cn(n1)c(C)c(c1C)N(C(F)F)S(=O)(=O)c(c(Cl)c2)c(Cl)cc2CCCOC[C@@H](CC34)N(CC3)CC4	0.39	6.409	68.12859	602.2812	1	0	2	2	1
33	Cn(n1)c(C)c(c1C)N(C(F)F)S(=O)(=O)c(c(Cl)c2)c(Cl)cc2CCCOC[C@H]3CCCN3	0.11	6.959	61.16283	542.3965	0	1	3	2	1
34	Cn(n1)c(C)c(c1C)N(C(F)F)S(=O)(=O)c(c(Cl)c2)c(Cl)cc2CCCOC[C@H]3CCCCN3	0.84	6.076	68.89843	564.8906	2	1	3	2	1
35	Cn(n1)c(C)c(c1C)N(C(F)F)S(=O)(=O)c(c(Cl)c2)c(Cl)cc2CCCOC[C@@H]3CCCCN3	0.57	6.244	68.63705	557.2879	2	1	3	2	1
36	CC(C)Cc1c(c(C)n(n1)C)NS(=O)(=O)c(cc2)ccc2-c(c3)ccc(c34)CCCN(C)C4	2	5.699	67.70174	237.517	6	0	1	3	0
37	CC(C)Cc1c(c(C)n(n1)C)N(C)S(=O)(=O)c(cc2)ccc2CCCN3CCN(C)CC3	1370	2.863	1.806114	103.6508	0	0	0	0	0
38	c1cncc(c1C)NS(=O)(=O)c2c(Cl)cc(cc2Cl)-c3cc(ncc3)N4CCNCC4	1500	2.824	76.5594	164.9332	5	0	0	3	0
39	Cn(n1)c(C)c(c1C)N(CC(F)F)S(=O)(=O)c(c(Cl)c2)c(Cl)cc2CCCN3CCN(C)CC3	1700	2.77	21.79909	302.5449	3	1	2	2	0
40	c1nccn1CCCCCCc2ccc(cc2)S(=O)(=O)N(C)c(c3C)c(C)n(n3)C	2100	2.678	81.45197	102.0582	2	0	0	2	0
41	Cn(n1)c(C)c(c1C)NS(=O)(=O)c2ccc(nc2)N(CC3)Cc(c34)cc(OC)c(c4)OC	2300	2.638	46.89117	256.3244	4	0	0	2	1
42	CC(C)Cc1c(c(C)n(n1)C)NS(=O)(=O)c(cc2)ccc2-c(ccc3)cc3CNCCc4ccccc4	2300	2.638	39.77681	203.7822	2	0	1	1	1

43	c1ccccc1CNc(nc2)ccc2S(=O)(=O)Nc(c3C)c(C)n(n3)C	17	4.77	4.345764	548.3713	3	0	2	1	0
44	c1c[nH]c(c12)ccc(c2)CNc(nc3)ccc3S(=O)(=O)Nc(c4C)c(C)n(n4)C	2.7	5.569	5.511337	586.9538	3	1	3	1	0
45	CC(C)Cc1c(c(C)n(n1)C)NS(=O)(=O)c(cc2)ccc2-c(ccc3)cc3CNC4CCCC4	2300	2.638	73.73226	290.4482	9	0	2	3	0
46	Cn(n1)c(C)c(c1C)N(CC(F)(F)F)S(=O)(=O)c(c(Cl)c2)c(Cl)cc2CCCN3CCN(C)CC3	2400	2.62	72.12158	101.1468	2	0	0	2	0
47	Cn(n1)c(C)c(c1C)NS(=O)(=O)c(cc2)ccc2-c(ccc3)cc3-c4nccn4C	2400	2.62	13.95073	373.499	4	0	1	2	0
48	Cn(n1)c(C)c(c1C)NS(=O)(=O)c(cc2)ccc2-c(ccc3)cc3C4=NC=NC4	2600	2.585	9.935379	338.855	4	0	3	2	0
49	c1nccn1CCCCCc2ccc(cc2)S(=O)(=O)N(C)c(c3C)c(C)n(n3)C	2900	2.538	34.35161	275.6621	4	0	0	2	0
50	Cn(n1)cc(c1C)NS(=O)(=O)c2c(Cl)cc(Br)cc2Cl	3360	2.474	8.909531	127.8979	0	0	1	1	0
51	CCCN(CCC)Cc1cc(ccc1)-c2cc(Cl)c(c(Cl)c2)S(=O)(=O)N(C)c(c3C)c(C)n(n3)C	3700	2.432	71.71079	209.6625	8	0	0	2	0
52	CC(C)Cc1c(c(C)n(n1)C)NS(=O)(=O)c(cc2)ccc2-c(ccc3)cc3CN(C4)CCC4N(C)C	3800	2.42	78.10366	273.5142	5	0	3	3	0
53	CC(C)Cc1c(c(C)n(n1)C)NS(=O)(=O)c(cc2)ccc2-c(ccc3)cc3CNCc4ccccc4	4000	2.398	68.47491	410.7389	7	0	2	3	0
54	Cn(n1)c(C)c(c1C)NS(=O)(=O)c2ccc(nc2)N(CC3)Cc(c34)cccc4N	4300	2.367	34.89803	234.3312	3	0	3	1	0
55	C1CCCCC1NCc2cc(ccc2)-c3cc(Cl)c(c(Cl)c3)S(=O)(=O)N(C)c(c4C)c(C)n(n4)C	4480	2.349	67.71539	237.0425	6	0	1	2	0
56	C1CN(C)CCN1CCCc2ccc(cc2)S(=O)(=O)N(C)c(c3C)c(C)n(n3)C	6100	2.215	46.56837	177.3745	4	0	0	2	0
57	Cn(n1)c(C)c(c1C)NS(=O)(=O)c(c2)ccc(c23)nc(cc3)NC4CCNCC4	6300	2.201	44.89307	227.4852	3	1	3	2	0
58	Cn(n1)c(C)c(c1C)NS(=O)(=O)c(cc2)ccc2-c3cc(ncc3)N4CCCCC4	9300	2.032	51.67931	231.04	4	0	1	2	0
59	C1CN(C)CCN1CCNc(nc2)ccc2S(=O)(=O)Nc(c3C)c(C)n(n3)C	8.2	5.086	30.92605	610.2317	1	0	2	1	0
60	C1CNCCN1CCNc(nc2)ccc2S(=O)(=O)Nc(c3C)c(C)n(n3)C	16	4.796	30.60973	576.6797	1	1	2	1	0

61	C1CCCCN1CCNc(nc2)ccc2S(=O)(=O)Nc(c3C)c(C)n(n3)C	74	4.131	35.70686	595.9395	3	0	2	1	0
62	C1CN(C)CCC1CCNc(nc2)ccc2S(=O)(=O)Nc(c3C)c(C)n(n3)C	1	6	31.03297	606.8787	2	0	2	1	0
63	C1CN(C)CCN1CCCNc(nc2)ccc2S(=O)(=O)Nc(c3C)c(C)n(n3)C	2.4	5.62	33.31258	633.0438	1	0	2	1	0
64	c1nccn1CCCNc(nc2)ccc2S(=O)(=O)Nc(c3C)c(C)n(n3)C	8.7	5.06	12.8813	549.934	1	0	2	1	0
65	Cn(n1)c(C)c(c1C)NS(=O)(=O)c2ccc(nc2)N(C3)CC[C@@H]3N4CCNCC4	1.6	5.796	39.76412	596.1537	1	1	3	1	0
66	Cn(n1)c(C)c(c1C)NS(=O)(=O)c2ccc(nc2)N(C3)CCC[C@H]3N4CCNCC4	0.11	6.959	47.40777	606.0688	1	1	3	1	0
67	C1CN(C)CCN1Cc2ccc(cc2)-c3cc(Cl)c(c(Cl)c3)S(=O)(=O)Nc(c4C)c(C)n(n4)C	4.2	5.377	45.7551	621.8898	2	0	1	2	0
68	CCN(CC)Cc1ccc(cc1)-c2cc(Cl)c(c(Cl)c2)S(=O)(=O)Nc(c3C)c(C)n(n3)C	45	4.347	45.51025	590.324	2	0	1	2	0
69	Cn(n1)c(C)c(c1C)NS(=O)(=O)c(c(Cl)c2)c(Cl)cc2-c3ccc(nc3)N4CCNCC4	0.75	6.125	36.14719	546.743	1	1	1	2	0
70	Cn(n1)c(C)c(c1C)NS(=O)(=O)c2c(Cl)cc(cc2Cl)-c3cccc(c34)CNCC4	4.3	5.367	45.02874	509.6245	1	1	2	2	0
71	C1CN(C)CCN1Cc2cc(ccc2)-c3cc(Cl)c(c(Cl)c3)S(=O)(=O)Nc(c4C)c(C)n(n4)C	0.007	8.155	41.52306	613.7082	2	0	1	2	0
72	CCN(CC)Cc1cc(ccc1)-c2cc(Cl)c(c(Cl)c2)S(=O)(=O)Nc(c3C)c(C)n(n3)C	0.12	6.921	43.36795	588.6692	2	0	1	2	0
73	Cn(n1)c(C)c(c1C)NS(=O)(=O)c(c(Cl)c2)c(Cl)cc2-c3cc(ncc3)N4CCNCC4	0.003	8.523	51.26844	503.4217	0	1	1	2	0
74	Cn1ncc(c1C)NS(=O)(=O)c(c(Cl)c2)c(Cl)cc2-c3cc(ncc3)N4CCNCC4	0.009	8.046	45.92805	485.1237	0	1	1	2	0
75	Cn(n1)cc(c1C)NS(=O)(=O)c(c(Cl)c2)c(Cl)cc2-c3cc(ncc3)N4CCNCC4	0.012	7.921	31.50211	524.2141	0	1	1	2	0
76	Cn(c1)ncc1NS(=O)(=O)c(c(Cl)c2)c(Cl)cc2-c3cc(ncc3)N4CCNCC4	0.059	7.229	40.18542	462.2958	0	1	1	2	0
77	CC(C)Cc1c(c(C)nn1C)NS(=O)(=O)c(c(Cl)c2)c(Cl)cc2-c3cc(ncc3)N4CCNCC4	0.025	7.602	65.73174	587.2233	1	1	1	3	0
78	CC(C)Cc1c(c(C)n(n1)C)NS(=O)(=O)c(c(Cl)c2)c(Cl)cc2-c3cc(ncc3)N4CCNCC4	0.004	8.398	65.88216	593.1953	1	1	1	3	0

79	Cn(n1)c(C)c(c1C)NS(=O)(=O)c(c(Cl)c2)c(Cl)cc2-c3cc(ccc3)N4CCNCC4	0.017	7.77	35.63739	547.7782	1	1	1	2	0
80	Cn(n1)c(C)c(c1C)NS(=O)(=O)c(c2)ccc(c23)nc(Cl)cc3	17700	1.752	3.955193	220.1181	3	0	1	2	0
81	Cn(n1)c(C)c(c1C)NS(=O)(=O)c2c(C)cc(OC)c(C)c2C	20000	1.699	20.0957	118.1765	0	0	1	1	0
82	Cc1c(C)c(OC)cc(C)c1S(=O)(=O)Nc(c2C)cccn2	70000	1.155	8.926231	196.4011	0	0	1	1	0
83	Cn1ncc(c1C)NS(=O)(=O)c2c(Cl)cc(Br)cc2Cl	70800	1.15	7.063606	133.0235	0	0	1	1	0
84	Cn(n1)c(C)c(c1C)NS(=O)(=O)c(c2F)ccc(Br)c2	87000	1.06	5.648079	155.2177	1	0	1	1	0
85	Cn(n1)c(C)c(c1C)NS(=O)(=O)c(c(Cl)c2)c(Cl)cc2-c3cc(ccc3)C4CCNCC4	0.023	7.638	41.86494	552.1442	1	1	2	2	2
86	Cn(n1)c(C)c(c1C)NS(=O)(=O)c(c(Cl)c2)c(Cl)cc2-c3cc(ccc3)N(CC4)CCN4C	0.025	7.602	35.35864	581.3237	1	0	1	2	0
87	Cn1cncc1C#Cc2cc(Cl)c(c(Cl)c2)S(=O)(=O)Nc(c3C)c(C)n(n3)C	17	4.77	2.616684	470.862	0	0	2	2	0
88	C1CN(C)CCN1CC#Cc2cc(Cl)c(c(Cl)c2)S(=O)(=O)Nc(c3C)c(C)n(n3)C	4.6	5.337	31.24922	563.2572	0	0	1	2	0
89	C1CN(C)CCN1CCCc2cc(Cl)c(c(Cl)c2)S(=O)(=O)Nc(c3C)c(C)n(n3)C	0.39	6.409	43.68302	569.3541	2	0	1	2	0
90	C1CN(C)CCN1CCCc2ccc(cc2)S(=O)(=O)Nc(c3C)c(C)n(n3)C	3.5	5.456	39.64335	631.9458	4	0	1	2	0
91	C1CN(C)CCC1CCCc2cc(Cl)c(c(Cl)c2)S(=O)(=O)Nc(c3C)c(C)n(n3)C	0.033	7.481	47.53413	574.1716	1	0	1	2	1
92	C1CN(C)CCC1CCCc2ccc(cc2)S(=O)(=O)Nc(c3C)c(C)n(n3)C	0.19	6.721	42.33545	637.5096	3	0	1	2	1
93	C1CNCCC1CCCc2cc(Cl)c(c(Cl)c2)S(=O)(=O)Nc(c3C)c(C)n(n3)C	0.008	8.097	50.82745	544.7672	1	1	2	2	1
94	C1CNCCC1CCCc2ccc(cc2)S(=O)(=O)Nc(c3C)c(C)n(n3)C	0.13	6.886	46.45477	608.4706	3	1	2	2	1
95	C1CN(C)CCC1CCCCc2cc(Cl)c(c(Cl)c2)S(=O)(=O)Nc(c3C)c(C)n(n3)C	0.004	8.398	48.47855	590.1843	1	0	1	2	2
96	C1CN(C)CCC1CCCCc2ccc(cc2)S(=O)(=O)Nc(c3C)c(C)n(n3)C	0.04	7.398	46.90226	661.8717	3	0	1	2	2

97	C1CNCCC1CCCCc2cc(Cl)c(c(Cl)c2)S(=O)(=O)Nc(c3C)c(C)n(n3)C	0.005	8.301	55.15211	568.7104	1	1	2	2	2
98	C1CNCCC1CCCCc2ccc(cc2)S(=O)(=O)Nc(c3C)c(C)n(n3)C	0.029	7.538	49.07575	632.0963	3	1	2	2	2
99	Cn(n1)c(C)c(c1C)NS(=O)(=O)c2c(Cl)cc(Br)cc2Cl	7.3	5.137	10.80876	284.9835	0	0	1	1	0
100	Cn(n1)c(C)c(c1C)N(C)S(=O)(=O)c2c(Cl)cc(Br)cc2Cl	3.58	5.446	20.92661	310.7569	0	0	0	1	0
101	Cn(n1)c(C)c(c1C)N(C)S(=O)(=O)c(c(Cl)c2)c(Cl)cc2-c3cc(ncc3)N4CCNCC4	0.004	8.398	41.17429	568.1613	0	1	0	2	0
102	Cn(n1)c(C)c(c1C)N(C)S(=O)(=O)c(cc2)ccc2-c3cc(ncc3)N4CCNCC4	0.022	7.658	37.92691	627.8909	2	1	0	2	0
103	C1CN(C)CCN1Cc2cc(ccc2)-c3ccc(cc3)S(=O)(=O)Nc(c4C)c(C)n(n4)C	0.009	8.046	37.53754	676.3737	4	0	1	2	0
104	C1CN(C)CCN1Cc2cc(ccc2)-c3ccc(cc3)S(=O)(=O)N(C)c(c4C)c(C)n(n4)C	0.017	7.77	46.5146	695.424	4	0	0	2	0
105	C1CN(C)CCN1CCCc2cc(Cl)c(c(Cl)c2)S(=O)(=O)N(C)c(c3C)c(C)n(n3)C	0.19	6.721	52.26467	595.0131	2	0	0	2	0
106	C1CN(C)CCN1CCCc2cc(Cl)c(c(Cl)c2)S(=O)(=O)N(CC)c(c3C)c(C)n(n3)C	0.37	6.432	61.48373	615.4217	2	0	0	2	0
107	c1nccn1CCCCCc2cc(Cl)c(c(Cl)c2)S(=O)(=O)N(C)c(c3C)c(C)n(n3)C	0.47	6.328	38.62635	565.9918	2	0	0	2	0
108	c1nccn1CCCCCCc2cc(Cl)c(c(Cl)c2)S(=O)(=O)N(C)c(c3C)c(C)n(n3)C	0.3	6.523	44.18472	598.0469	2	0	0	2	1
109	C1CN(C)CCC1CCCc2cc(Cl)c(c(Cl)c2)S(=O)(=O)N(C)c(c3C)c(C)n(n3)C	0.018	7.745	58.02909	599.8386	1	0	0	2	1

110	C1CN(C)CCC1CCCc2ccc(cc2)S(=O)(=O)N(C)c(c3C)c(C)n(n3)C	0.39	6.409	50.52856	655.5903	3	0	0	2	1
111	Cn(n1)c(C)c(c1C)NS(=O)(=O)c(c2C)ccc(Br)c2	107000	0.971	12.25186	152.2567	1	0	1	1	0
112	C1CNCCC1CCCc2cc(Cl)c(c(Cl)c2)S(=O)(=O)N(C)c(c3C)c(C)n(n3)C	4	5.398	73.86381	121.2472	1	1	1	2	1
113	CC(C)Cc1c(c(C)n(n1)C)NS(=O)(=O)c(cc2)ccc2-c(cc3)cc(c34)CNCCC4	4	5.398	68.61145	238.6252	6	1	2	3	0
114	C1CN(C)CCC1Cc2cc(ccc2)-c3ccc(cc3)S(=O)(=O)Nc(c4C)c(C)n(n4)C	5	5.301	43.59296	295.8383	3	0	1	2	3
115	C1CN(C)CCN1Cc2cc(ccc2)-c3cc(Cl)c(c(Cl)c3)S(=O)(=O)N(C)c(c4C)c(C)n(n4)C	5	5.301	69.34592	206.3305	2	0	0	2	0
116	Cn(n1)c(C)c(c1C)N(CC)S(=O)(=O)c(c(Cl)c2)c(Cl)cc2-c3cc(ncc3)N4CCNCC4	3	5.523	58.14439	205.6742	0	1	0	2	0
117	CC(C)Cc1c(c(C)n(n1)C)NS(=O)(=O)c(cc2)ccc2-c(ccc3)cc3CNCCc4cccnc4	6	5.222	58.9314	404.9705	6	0	2	3	0
118	C1CN(C)CCC1CCCc2cc(Cl)c(c(Cl)c2)S(=O)(=O)N(CC)c(c3C)c(C)n(n3)C	0.024	7.62	64.30871	621.1469	1	0	0	2	1
119	Cn(n1)c(C)c(c1C)N(C(F)F)S(=O)(=O)c(c(Cl)c2)c(Cl)cc2CCCC3CCN(C)CC3	0.012	7.921	62.31933	557.1006	1	0	0	2	1
120	Cn(n1)c(C)c(c1C)N(CC(F)F)S(=O)(=O)c(c(Cl)c2)c(Cl)cc2CCCC3CCN(C)CC3	0.18	6.745	73.94154	578.1528	1	0	0	2	1
121	Cn(n1)c(C)c(c1C)N(CC(F)(F)F)S(=O)(=O)c(c(Cl)c2)c(Cl)cc2CCCC3CCN(C)CC3	0.25	6.602	73.64769	547.157	1	0	0	2	1

122	Cn(n1)c(C)c(c1C)N(C(F)F)S(=O)(=O)c(c(Cl)c2)c(Cl)cc2CCCC3CCNCC3	0.009	8.046	63.10323	527.247	1	1	1	2	1
123	Cn(n1)c(C)c(c1C)N(C(F)F)S(=O)(=O)c(c(Cl)c2)c(Cl)cc2CCCN3CCN(C)CC3	0.16	6.796	55.56719	549.5851	2	0	0	2	0
124	Cn(n1)c(C)c(c1C)N(C(F)F)S(=O)(=O)c(c(Cl)c2)c(Cl)cc2CCCCC3CCNCC3	0.004	8.398	69.58496	565.3823	1	1	1	2	2
125	Cn(n1)c(C)c(c1C)N(C(F)F)S(=O)(=O)c(c(Cl)c2)c(Cl)cc2CCCCC3CCN(C)CC3	0.005	8.301	66.99833	593.6801	1	0	0	2	2
126	Cn(n1)c(C)c(c1C)N(C(F)F)S(=O)(=O)c(c(Cl)c2)c(Cl)cc2CCCOC3CCN(C)CC3	0.005	8.301	62.40276	582.7038	1	0	0	2	2
127	Cn(n1)c(C)c(c1C)N(C(F)F)S(=O)(=O)c(c(Cl)c2)c(Cl)cc2CCCCCCN(C)C	0.008	8.097	57.41154	575.5027	2	0	0	2	1
128	Cn(n1)c(C)c(c1C)N(C(F)F)S(=O)(=O)c(c(Cl)c2)c(Cl)cc2CCCOC[C@H]3CCCN3C	0.074	7.131	60.88722	590.5065	0	0	2	2	1
129	Cn(n1)c(C)c(c1C)N(C(F)F)S(=O)(=O)c(c(Cl)c2)c(Cl)cc2CCCOC[C@H]3CCCCN3C	0.28	6.553	69.37699	603.5585	2	0	2	2	1
130	Cn(n1)c(C)c(c1C)N(C(F)F)S(=O)(=O)c(c(Cl)c2)c(Cl)cc2CCCOC[C@@H]3CCCN3C	0.045	7.347	61.55376	589.501	0	0	2	2	1
131	Cn(n1)c(C)c(c1C)N(C(F)F)S(=O)(=O)c(c(Cl)c2)c(Cl)cc2CCCOC[C@@H]3CCCCN3C	0.15	6.824	65.43075	607.4633	2	0	2	2	1
132	Cn(n1)c(C)c(c1C)N(C(F)F)S(=O)(=O)c(c(Cl)c2)c(Cl)cc2CCCN(C3)CCN([C@@H]34)CCC4	0.047	7.328	65.84558	592.1008	2	0	2	2	0
133	Cn(n1)c(C)c(c1C)NS(=O)(=O)c(cc2)ccc2-c3cc(ncc3)N(CC4C)CCN4	8	5.097	49.37895	264.9967	2	1	4	2	0

134	Cn(n1)c(C)c(c1C)NS(=O)(=O)c(cc2)ccc2-c3cc(ncc3)N4CCCNCC4	8	5.097	51.98119	257.4642	2	1	2	2	0
135	C1CNCCC1CCCc2cc(Cl)c(c(Cl)c2)S(=O)(=O)N(CC)c(c3C)c(C)n(n3)C	9	5.046	71.12009	131.4874	1	1	1	2	1
136	CC(C)Cc1c(c(C)n(n1)C)NS(=O)(=O)c(cc2)ccc2-c(c3)cc(C)cc3CN(C)C	560	3.252	67.908	230.7429	5	0	1	3	0
137	C1CN(C)CCC1Cc2cc(ccc2)-c3ccc(cc3)S(=O)(=O)N(C)c(c4C)c(C)n(n4)C	9	5.046	65.69802	259.8842	3	0	0	2	3
138	CC(C)Cc1c(c(C)n(n1)C)NS(=O)(=O)c(c(Cl)c2)c(Cl)cc2CCCC3CCNCC3	10	5	61.53657	150.4742	2	1	2	3	1
139	Cn(n1)c(C)c(c1C)NS(=O)(=O)c2ccc(nc2)N(C3)CCCC3N4CCNCC4	10	5	58.86944	148.4422	1	1	3	1	0
140	CC(C)Cc1c(c(C)n(n1)C)NS(=O)(=O)c(cc2)ccc2-c(c3)ccc(Cl)c3CN(C)C	10	5	64.60429	240.5431	4	0	1	3	0
141	C1CNCCCN1CCCc2cc(Cl)c(c(Cl)c2)S(=O)(=O)N(C)c(c3C)c(C)n(n3)C	11	4.959	68.64092	111.4038	2	1	1	2	0
142	CC(C)Cc1c(c(C)n(n1)C)NS(=O)(=O)c(cc2)ccc2-c(c3)ccc(F)c3CN(C)C	11	4.959	67.09584	238.6278	4	0	1	3	0
143	CC(C)Cc1c(c(C)n(n1)C)NS(=O)(=O)c(cc2)ccc2-c(cc3)cc(c34)CNCC4	11	4.959	64.07021	247.8613	4	1	2	3	0
144	Cn(n1)c(C)c(c1C)N(C(F)F)S(=O)(=O)c(cc2)ccc2-c3cc(ncc3)N4CCNCC4	12	4.921	55.60858	239.5956	2	1	0	2	0
145	CC(C)Cc1c(c(C)n(n1)C)NS(=O)(=O)c(cc2)ccc2-c(c3)ccc(C)c3CN(C)C	13	4.886	72.78844	236.9129	4	0	1	3	0

146	CC(C)Cc1c(c(C)n(n1)C)NS(=O)(=O)c(cc2)ccc2-c(ccc3)cc3CN(C)C	14	4.854	60.99546	270.2574	5	0	1	3	0
147	CC(C)Cc1c(c(C)n(n1)C)NS(=O)(=O)c(c(Cl)c2)c(Cl)cc2CCCC3CCN(C)CC3	16	4.796	76.63618	132.6045	2	0	1	3	1
148	Cn(n1)c(C)c(c1C)N(C(F)F)S(=O)(=O)c(c(Cl)c2)c(Cl)cc2CCCOC(C34)CN(CC3)CC4	17	4.77	71.7451	110.0306	1	0	2	2	1
149	CC(C)c1c(c(C(C)C)n(n1)C)NS(=O)(=O)c(cc2)ccc2CCCC3CCN(C)CC3	620	3.208	69.63501	201.5527	15	0	1	6	1
150	CN(C)Cc1cc(ccc1)-c(c2C(F)(F)F)ccc(c2)S(=O)(=O)Nc(c(C)n(n3)C)c3CC(C)C	17	4.77	82.40714	211.2225	5	0	1	3	0
151	CC(C)(N)c1cc(ccc1)-c2ccc(cc2)S(=O)(=O)Nc(c(C)n(n3)C)c3CC(C)C	18	4.745	66.33306	247.1205	5	0	3	3	0
152	Cn(n1)c(C)c(c1C)N(C(F)F)S(=O)(=O)c(c(Cl)c2)c(Cl)cc2-c3cc(ncc3)N4CCNCC4	18	4.745	47.77505	222.3117	0	1	0	2	0
153	CC(C)Cc1c(c(C)n(n1)C)NS(=O)(=O)c(cc2)ccc2-c(ccc3)cc3CNC	19	4.721	59.11067	279.6383	5	0	2	3	0
154	CC(C)Cc1c(c(C)n(n1)C)N(C(F)F)S(=O)(=O)c(c(Cl)c2)c(Cl)cc2-c3cc(ncc3)N4CCNCC4	19	4.721	76.22842	225.2002	1	1	0	3	0
155	C1CN(C)CCC1CCCc2ccc(cc2)S(=O)(=O)Nc(c(C)n(n3)C)c3CCc4ccccc4	630	3.201	52.86696	340.679	5	0	1	3	3
156	Cn(n1)c(C)c(c1C)N(CC(F)F)S(=O)(=O)c(cc2)ccc2-c3cc(ncc3)N4CCNCC4	19	4.721	51.82272	269.4833	2	1	0	2	0
157	C1CC1Cc2c(c(C)n(n2)C)NS(=O)(=O)c(cc3)ccc3-c(ccc4)cc4CN(C)C	19	4.721	45.13498	287.819	5	0	1	3	0

158	c1ccnc(C)c1COc(cc2)ccc2-c3cc(ccc3)C4CCNCC4	0.3	6.523	25.91278	648.0239	4	1	1	0	4
159	c1ccnc(C)c1COc2c(Cl)cc(cc2Cl)-c3cc(ncc3)N4CCNCC4	0.3	6.523	23.95212	542.08	3	1	0	0	2
160	c1ccnc(C)c1COc(cc2)ccc2-c3cc(ncc3)N4CCNCC4	0.09	7.046	14.83902	621.1462	3	1	0	0	2
161	c1ccnc(C)c1COc(c(F)c2)cc(F)c2-c3cc(ncc3)N4CCNCC4	0.01	8	19.33319	559.3447	3	1	0	0	2
162	c1ccnc(C)c1COc(cc2)cc(F)c2-c3cc(ncc3)N4CCNCC4	0.06	7.222	16.94582	586.9478	3	1	0	0	2
163	c1ccnc(C)c1COc(cc2F)cc(F)c2-c3cc(ncc3)N4CCNCC4	0.03	7.523	21.85158	572.6468	3	1	0	0	2
164	c1ccnc(C)c1COc(c2)ccc(c2C)-c3cc(ncc3)N4CCNCC4	0.08	7.097	32.31247	642.6124	3	1	0	0	3
165	c1ccnc(C)c1COc(cc2C)cc(C)c2-c3cc(ncc3)N4CCNCC4	1.8	5.745	41.07552	660.1984	3	1	0	0	4
166	c1ncccc1COc2c(Cl)cc(cc2Cl)-c3cc(ncc3)N4CCNCC4	0.1	7	19.99075	512.1008	3	1	0	0	2
167	n1ccccc1COc2c(Cl)cc(cc2Cl)-c3cc(ncc3)N4CCNCC4	6.5	5.187	17.6275	516.0939	1	1	2	0	0
168	c1cc(C)nc(C)c1COc2c(Cl)cc(cc2Cl)-c3cc(ncc3)N4CCNCC4	1.3	5.886	32.46057	587.5889	3	1	0	0	2
169	Cc1nccc(C)c1COc2c(Cl)cc(cc2Cl)-c3cc(ncc3)N4CCNCC4	0.2	6.699	27.86815	565.1998	2	1	0	0	2

170	c1ncccc1CCOc2c(Cl)cc(cc2Cl)-c3cc(ncc3)N4CCNCC4	0.5	6.301	28.03131	541.0079	3	1	0	0	3
171	c1cnccc1CCOc2c(Cl)cc(cc2Cl)-c3cc(ncc3)N4CCNCC4	0.7	6.155	27.64124	541.3595	0	1	0	0	2
172	n1oc(C)cc1COc2c(Cl)cc(cc2Cl)-c3cc(ncc3)N4CCNCC4	4.4	5.357	33.49242	488.6071	3	1	2	0	0
173	Cc(s1)nc(C)c1COc2c(Cl)cc(cc2Cl)-c3cc(ncc3)N4CCNCC4	0.08	7.097	34.84499	523.6876	2	1	0	2	2
174	Cc(o1)nc(C)c1COc2c(Cl)cc(cc2Cl)-c3cc(ncc3)N4CCNCC4	0.03	7.523	34.61153	535.3878	2	1	0	0	2
175	Cc(n1)scc1COc2c(Cl)cc(cc2Cl)-c3cc(ncc3)N4CCNCC4	15	4.824	34.93798	496.5437	0	1	2	1	0
176	s1ccnc1COc2c(Cl)cc(cc2Cl)-c3cc(ncc3)N4CCNCC4	14	4.854	23.51495	466.6402	0	1	2	2	0
177	CC(C)Cc1c(c(C)n(n1)C)NS(=O)(=O)c(cc2)ccc2-c(c3)ccc(c34)CCN(C)C4	20	4.699	65.87747	235.7951	4	0	1	3	0
178	Cn(n1)c(C)c(c1C)N(CC(F)(F)F)S(=O)(=O)c(cc2)ccc2-c3cc(ncc3)N4CCNCC4	21	4.678	63.33225	240.6435	2	1	0	2	0
179	CC(C)Cc1c(c(C)n(n1)C)NS(=O)(=O)c(cc2)ccc2-c(ccc3)cc3CNCCC	21	4.678	71.46444	278.3194	8	0	2	3	0
180	CC(C)Cc1c(c(C)n(n1)C)NS(=O)(=O)c(cc2)ccc2-c(ccc3)cc3CN(C)CC	21	4.678	49.36372	277.0918	5	0	1	3	0
181	CN(C)CCCCCCCCc1ccc(cc1)S(=O)(=O)N(C)c(c2C)c(C)n(n2)C	21	4.678	75.00762	157.5272	4	0	0	2	2

182	n1oc(C)c(c1C)NS(=O)(=O)c(cc2)ccc2-c3cc(ncc3)N4CCNCC4	650	3.187	28.78793	275.0196	5	1	1	1	0
183	CC(C)Cc1c(c(C)n(n1)C)NS(=O)(=O)c(cc2)ccc2-c(ccc3)cc3CNCC	22	4.658	63.10139	276.4001	5	0	2	3	0
184	CC(C)Cc1c(c(C)n(n1)C)NS(=O)(=O)c(cc2)ccc2-c(ccc3)cc3CC4CCNCC4	25	4.602	74.10116	275.3166	4	1	2	3	3
185	CC(C)Cc1c(c(C)n(n1)C)NS(=O)(=O)c(cc2)ccc2-c(c(F)cc3)cc3CN(C)C	29	4.538	70.36327	237.2983	5	0	1	3	0
186	C1CCCN1C(C)(C)c2cc(ccc2)-c3ccc(cc3)S(=O)(=O)Nc(c(C)n(n4)C)c4CC(C)C	30	4.523	59.40109	278.8518	5	0	1	3	0
187	CC(C)Cc1c(c(C)n(n1)C)NS(=O)(=O)c(cc2)cc(Cl)c2-c(ccc3)cc3CN(C)C	33	4.481	60.65449	257.7782	5	0	1	3	0
188	Cn(n1)c(C)c(c1C)N(CC(F)F)S(=O)(=O)c(c(Cl)c2)c(Cl)cc2CCCCC3CCNCC3	33	4.481	72.60497	140.8702	1	1	1	2	2
189	Cn(n1)c(C)c(c1C)N(CC(F)F)S(=O)(=O)c(c(Cl)c2)c(Cl)cc2CCCOC3CCN(C)CC3	34	4.469	73.99207	128.7649	1	0	0	2	2
190	CN(C)CCCCCCCc1ccc(cc1)S(=O)(=O)N(C)c(c2C)c(C)n(n2)C	36	4.444	59.16158	159.1327	4	0	0	2	2
191	CC(C)Cc1c(c(C)n(n1)C)N(C)S(=O)(=O)c(cc2)ccc2-c(c3)ccc(c34)CCNC4	37	4.432	70.10909	249.2958	4	1	1	3	0
192	CCCc1c(c(C)n(n1)C)NS(=O)(=O)c(cc2)ccc2-c(ccc3)cc3CN(C)C	39	4.409	45.07443	282.4969	6	0	1	3	0
193	Cn(n1)c(C)c(c1C)NS(=O)(=O)c(cc2)ccc2-c3cc(ncc3)N(CC4C)CC(C)N4	40	4.398	52.44183	281.0067	2	1	6	2	0

194	CC(C)Cc1c(c(C)n(n1)C)NS(=O)(=O)c(c2)ccc(c2C)-c(ccc3)cc3CN(C)C	41	4.387	72.94358	237.5067	5	0	1	3	0
195	Cn(n1)c(C)c(c1C)N(CC(F)(F)F)S(=O)(=O)c(c(Cl)c2)c(Cl)cc2CCCOC3CCN(C)CC3	46	4.337	78.91819	110.5059	1	0	0	2	2
196	CC(C)Cc1c(c(C)n(n1)C)NS(=O)(=O)c(cc2)ccc2-c(ccc3)cc3CN(CC)CC	47	4.328	76.38846	257.7814	5	0	1	3	0
197	C1CNCCC1Cc2cc(ccc2)-c3ccc(cc3)S(=O)(=O)N(C)c(c4C)c(C)n(n4)C	47	4.328	62.04714	267.6076	3	1	1	2	3
198	CCCCc1c(c(C)n(n1)C)NS(=O)(=O)c(cc2)ccc2-c(ccc3)cc3CN(C)C	47	4.328	44.13707	284.9752	6	0	1	3	0
199	C1CNCCN1CCCc2cc(Cl)c(c(Cl)c2)S(=O)(=O)N(C)c(c3C)c(C)n(n3)C	51	4.292	60.27339	123.381	2	1	0	2	0
200	CN(C)C(C)(C)c1cc(ccc1)-c2ccc(cc2)S(=O)(=O)Nc(c(C)n(n3)C)c3CC(C)C	51	4.292	69.42155	253.2519	5	0	1	3	0
201	C1CN(C)CCC1OCCCc2ccc(cc2)S(=O)(=O)Nc(c3C)c(C)n(n3)C	52	4.284	66.33344	158.8982	3	0	1	2	2
202	CC(C)Cc1c(c(C)n(n1)C)NS(=O)(=O)c(cc2)ccc2-c3cc(ccc3)C4CCNCC4	54	4.268	72.96918	277.1852	4	1	2	3	2
203	C1CN(C)CCN1CCCc2cc(Cl)c(cc2)S(=O)(=O)Nc(c3C)c(C)n(n3)C	740	3.131	54.05126	134.7111	3	0	1	2	0
204	CC(C)Cc1c(c(C)n(n1)C)NS(=O)(=O)c(cc2)ccc2CCCCC3CCNCC3	56	4.252	91.32825	166.7409	4	1	2	3	2
205	CC(C)Cc1c(c(C)n(n1)C)NS(=O)(=O)c(cc2)ccc2-c(c3)ccc(F)c3CN4CCCC4	57	4.244	75.1299	236.2391	4	0	1	3	0

206	CC(C)Cc1c(c(C)n(n1)C)NS(=O)(=O)c(cc2)c(Cl)cc2-c(ccc3)cc3CN(CC)CC	58	4.237	55.4846	265.1255	4	0	1	3	0
207	Cn(n1)c(C)c(c1C)N(CC(F)F)S(=O)(=O)c(c(Cl)c2)c(Cl)cc2CCCCC3CCN(C)CC3	60	4.222	82.30477	111.408	1	0	0	2	2
208	CC(C)Cc1c(c(C)n(n1)C)NS(=O)(=O)c(cc2)ccc2-c(ccc3)cc3CN4CCCC4C	61	4.215	75.16734	278.5908	5	0	1	3	0
209	CN(C)CCc1cc(ccc1)-c2ccc(cc2)S(=O)(=O)Nc(c3C)c(C)n(n3)C	64	4.194	43.15472	261.4579	2	0	1	2	1
210	CN(C)Cc1c(OC(F)(F)F)ccc(c1)-c2ccc(cc2)S(=O)(=O)Nc(c(C)n(n3)C)c3CC(C)C	740	3.131	73.74663	220.222	4	0	1	3	0
211	Cn(n1)c(C)c(c1C)N(CC(F)(F)F)S(=O)(=O)c(c(Cl)c2)c(Cl)cc2CCCCC3CCNCC3	71	4.149	87.99103	106.2891	1	1	1	2	2
212	Cn(n1)c(C)c(c1C)N(CC(F)(F)F)S(=O)(=O)c(cc2)ccc2-c(ccc3)cc3CN4CCN(C)CC4	75	4.125	70.04358	239.1128	4	0	0	2	0
213	CC(C)Cc1c(c(C)n(n1)C)NS(=O)(=O)c(cc2)ccc2-c(ccc3)cc3CN4CCC4	76	4.119	67.84647	269.1054	5	0	1	3	0
214	CN(C)CCCCCCc1ccc(cc1)S(=O)(=O)N(C)c(c2C)c(C)n(n2)C	76	4.119	48.13432	174.6689	4	0	0	2	1
215	n1cscc1COc2c(Cl)cc(cc2Cl)-c3cc(ncc3)N4CCNCC4	9.9	5.004	28.37576	451.3828	0	1	2	1	0
216	s1cncc1COc2c(Cl)cc(cc2Cl)-c3cc(ncc3)N4CCNCC4	0.9	6.046	23.5122	457.1634	2	1	0	2	2
217	CCc(s1)nc(C)c1COc2c(Cl)cc(cc2Cl)-c3cc(ncc3)N4CCNCC4	0.3	6.523	41.52137	569.1482	5	1	0	2	2

218	CC(C)c(s1)nc(C)c1COc(c(Cl)c2)c(Cl)cc2-c3cc(ncc3)N4CCNCC4	3.8	5.42	45.74059	599.6309	8	1	0	2	2
219	C1CN(C)CCC1CCCCc2cc(Cl)c(c(Cl)c2)OCc3c(C)nccc3	1	6	41.63522	598.0634	4	0	0	0	3
220	C1COCCN1c(nccc2)c2COc3c(Cl)cc(cc3Cl)-c4cc(ncc4)N5CCNCC5	0.04	7.398	39.80682	611.2458	3	1	0	0	2
221	c1ccnc(CC(C)C)c1COc2c(Cl)cc(cc2Cl)-c3cc(ncc3)N4CCNCC4	0.04	7.398	37.91563	618.3062	4	1	0	0	2
222	c1ccccc1-c(nccc2)c2COc3c(Cl)cc(cc3Cl)-c4cc(ncc4)N5CCNCC5	0.2	6.699	28.04152	626.6342	5	1	0	0	2
223	c1ccnc(CCC)c1COc2c(Cl)cc(cc2Cl)-c3cc(ncc3)N4CCNCC4	0.04	7.398	38.40419	597.5712	5	1	0	0	2
224	CC(C)CCc(nccc1)c1COc2c(Cl)cc(cc2Cl)-c3cc(ncc3)N4CCNCC4	0.18	6.745	44.05565	644.4246	5	1	0	0	2
225	CC(C)Cc1c(c(C)n(n1)C)NS(=O)(=O)c(cc2)ccc2-c(ccc3)cc3CNCCOC	77	4.114	66.3436	279.9915	5	0	2	3	0
226	n1cccc(c1C)NS(=O)(=O)c2c(Cl)cc(cc2Cl)-c3cc(ncc3)N4CCNCC4	80	4.097	22.71613	309.8413	0	1	1	2	0
227	Cn(n1)c(C)c(c1C)N(CC(F)F)S(=O)(=O)c(c(Cl)c2)c(Cl)cc2CCCN3CCCNCC3	81	4.092	79.28284	117.1015	2	1	1	2	0
228	CC(C)Cc1c(c(C)n(n1)C)NS(=O)(=O)c(cc2)ccc2CCCC3CCNCC3	88	4.056	60.26726	206.1616	4	1	2	3	1
229	CN(C)CCCCCc1ccc(cc1)S(=O)(=O)N(C)c(c2C)c(C)n(n2)C	760	3.119	43.07949	173.7338	4	0	0	2	0

230	CC(C)Cc1c(c(C)n(n1)C)NS(=O)(=O)c(cc2)ccc2-c(c(Cl)cc3)cc3CN(C)C	94	4.027	70.57327	233.57	5	0	1	3	0
231	c1ccccc1CCc2c(c(C)n(n2)C)NS(=O)(=O)c(cc3)ccc3-c(ccc4)cc4CN(C)C	96	4.018	56.17418	411.6329	6	0	1	3	2
232	Cn(n1)c(C)c(c1C)N(C(F)F)S(=O)(=O)c(cc2)ccc2-c3cc(ccc3)C4CCNCC4	100	4	58.50918	265.3134	3	1	1	2	2
233	Cn(n1)c(C)c(c1C)N(CC(F)F)S(=O)(=O)c(c(Cl)c2)c(Cl)cc2CCCN3CCCN(C)CC3	101	3.996	86.14289	101.8907	2	0	0	2	0
234	Cn(n1)c(C)c(c1C)N(CC(F)(F)F)S(=O)(=O)c(c(Cl)c2)c(Cl)cc2CCCCC3CCN(C)CC3	110	3.959	81.77246	106.4484	1	0	0	2	2
235	c1ccccc1Cc2c(c(C)n(n2)C)NS(=O)(=O)c(cc3)ccc3-c(ccc4)cc4CN(C)C	110	3.959	59.73143	378.1173	4	0	1	3	1
236	CC(C)Cc1c(c(C)n(n1)C)NS(=O)(=O)c(cc2)ccc2CCCC3CCN(C)CC3	120	3.921	73.77403	174.1571	4	0	1	3	1
237	CC(C)Cc1c(c(C)n(n1)C)NS(=O)(=O)c(cc2)ccc2-c(ccc3)cc3CN4CCCC4	120	3.921	79.22675	263.3907	5	0	1	3	0
238	Cn(n1)c(C)c(c1C)N(CC(F)(F)F)S(=O)(=O)c(c(Cl)c2)c(Cl)cc2CCCC3CCNCC3	134	3.873	76.91984	119.9372	1	1	1	2	1
239	C1CN(C)CCC1CCCCc2ccc(cc2)S(=O)(=O)Nc(c(C)n(n3)C)c3CCC	140	3.854	75.84563	167.1891	5	0	1	3	2
240	CC1CN(CC(C)N1)CCCc2cc(Cl)c(c(Cl)c2)S(=O)(=O)N(C)c(c3C)c(C)n(n3)C	141	3.851	55.73635	159.0479	2	1	5	2	0
241	CC(C)Cc1c(c(C)n(n1)C)N(C)S(=O)(=O)c(cc2)ccc2-c(ccc3)cc3CN(CC)CC	144	3.842	77.13441	260.8699	5	0	0	3	0

242	Cn(n1)c(C)c(c1C)N(CC(F)F)S(=O)(=O)c(c(Cl)c2)c(Cl)cc2CCCC3CCNCC3	148	3.83	73.26562	134.4549	1	1	1	2	1
243	C1CN(C)CCC1CCCc2ccc(cc2)S(=O)(=O)Nc(c(C)n(n3)C)c3CC4CC4	150	3.824	63.20828	192.8222	4	0	1	3	1
244	CC(C)Cc1c(c(C)n(n1)C)NS(=O)(=O)c(cc2)ccc2-c(ccc3)cc3CNCCc4ccccn4	769	3.114	80.95477	353.2678	6	0	2	3	0
245	C1CN(C)CCN1CCCc2cc(Cl)c(c(Cl)c2)S(=O)(=O)N(C)c(c3CC)c(CC)n(n3)C	150	3.824	79.77478	106.1748	8	0	0	4	0
246	CC(C)Cc1c(c(C)n(n1)C)NS(=O)(=O)c(cc2)ccc2-c(ccc3)c(F)c3CN(C)C	153	3.815	46.55228	284.5712	4	0	1	3	0
247	C1CNCCC1CCCc2ccc(cc2)S(=O)(=O)N(C)c(c3C)c(C)n(n3)C	164	3.785	56.85882	189.0327	3	1	1	2	1
248	CC(C)Cc1c(c(C)n(n1)C)NS(=O)(=O)c(cc2)ccc2CCCOC3CCN(C)CC3	168	3.775	74.06911	180.0628	4	0	1	3	2
249	c1cnc(C)c(c1C)NS(=O)(=O)c2c(Cl)cc(cc2Cl)-c3cc(ncc3)N4CCNCC4	770	3.114	27.9232	287.3204	3	1	1	2	0
250	CC(C)Cc1c(c(C)n(n1)C)NS(=O)(=O)c(cc2)ccc2CCCCC3CCN(C)CC3	173	3.762	78.09805	180.4367	4	0	1	3	2
251	CC(C)Cc1c(c(C)n(n1)C)NS(=O)(=O)c(cc2)ccc2-c(c3CN(C)C)cccc3	174	3.759	60.04465	282.6362	4	0	1	3	0
252	CC(C)Cc1c(c(C)n(n1)C)NS(=O)(=O)c(c(Cl)c2)c(Cl)cc2CCCN3CCN(C)CC3	180	3.745	58.51835	131.5423	3	0	1	3	0
253	C1CN(C)CCN1CCc2cc(ccc2)-c3ccc(cc3)S(=O)(=O)Nc(c4C)c(C)n(n4)C	180	3.745	38.07765	300.3018	2	0	1	2	1

254	CC(C)Cc1c(c(C)n(n1)C)N(C)S(=O)(=O)c(cc2)ccc2-c3cc(ccc3)C4CCNCC4	190	3.721	74.25804	270.0796	4	1	1	3	2
255	CC(C)c1c(c(C(C)C)n(n1)C)NS(=O)(=O)c(cc2)ccc2-c(ccc3)cc3CN(C)C	200	3.699	68.18542	270.7691	16	0	1	6	0
256	Cn(n1)c(C)c(c1C)N(CC(F)(F)F)S(=O)(=O)C(C(Cl)C2)=C(Cl)C=C2CCCN3CCCNCC3	210	3.678	75.6782	89.12471	3	1	1	2	0
257	Cn(n1)c(C)c(c1C)N(CC(F)(F)F)S(=O)(=O)c(c(Cl)c2)c(Cl)cc2CCCN3CCCN(C)CC3	210	3.678	75.08932	103.1279	2	0	0	2	0
258	C1CN(C)CCC1CCCc2ccc(cc2)S(=O)(=O)Nc(c(C)n(n3)C)c3CCC	220	3.658	61.20754	176.8302	5	0	1	3	1
259	C1CN(C)CCC1CCCc2ccc(cc2)S(=O)(=O)Nc(c(C)n(n3)C)c3Cc4ccccc4	233	3.633	47.52406	336.271	3	0	1	3	2
260	CC(C)Cc1c(c(C)n(n1)C)N(C)S(=O)(=O)c(cc2)ccc2CCCC3CCN(C)CC3	234	3.631	80.20295	171.7471	4	0	0	3	1
261	CC(C)Cc1c(c(C)n(n1)C)NS(=O)(=O)c(cc2)ccc2-c(ccc3)cc3CNC(C)C	240	3.62	68.60212	295.7731	5	0	2	3	0
262	CC(C)C1CN(CCN1)c(ncc2)cc2-c3ccc(cc3)S(=O)(=O)Nc(c4C)c(C)n(n4)C	240	3.62	36.09697	311.1742	8	1	4	2	0
263	CC(C)Cc1c(c(C)n(n1)C)NS(=O)(=O)c(cc2)ccc2-c(ccc3)cc3CN4CCN(C)CC4	250	3.602	57.3913	293.8418	5	0	1	3	0
264	C1CN(C)CCC1CCC#Cc2ccc(cc2)S(=O)(=O)Nc(c3C)c(C)n(n3)C	250	3.602	29.51228	233.5634	3	0	1	2	2
265	NCc1cc(ccc1)-c2ccc(cc2)S(=O)(=O)Nc(c3C)c(C)n(n3)C	272	3.565	20.276	286.6542	4	0	3	2	0

266	CCN(CC)Cc1cc(ccc1)-c2cc(Cl)c(c(Cl)c2)S(=O)(=O)N(C)c(c3C)c(C)n(n3)C	310	3.509	60.49903	211.0646	2	0	0	2	0
267	CC(C)Cc1c(c(C)n(n1)C)NS(=O)(=O)c(cc2)ccc2-c(cc3)ccc3CN(C)C	314	3.503	63.2268	264.4539	5	0	1	3	0
268	CCCCc1c(c(C)n(n1)C)NS(=O)(=O)c(cc2)ccc2CCCC3CCN(C)CC3	330	3.481	61.60043	182.9346	5	0	1	3	1
269	CC(C)Cc1c(c(C)n(n1)C)NS(=O)(=O)c(cc2)ccc2-c(ccc3)cc3CNCc4cccnc4	330	3.481	77.68639	374.1048	6	0	2	3	0
270	CC(C)Cc1c(c(C)n(n1)C)NS(=O)(=O)c(c(Cl)c2)c(Cl)cc2-c(ccc3)cc3CN(CC)CC	810	3.092	71.69453	217.2009	3	0	1	3	0
271	c1nccn1CCCCCCCc2ccc(cc2)S(=O)(=O)N(C)c(c3C)c(C)n(n3)C	370	3.432	43.73984	276.2953	4	0	0	2	2
272	C1CN(C)CCC1CCCCc2ccc(cc2)S(=O)(=O)Nc(c(C)n(n3)C)c3CCCC	400	3.398	82.40956	158.0999	5	0	1	3	2
273	CC(C)Cc1c(c(C)n(n1)C)NS(=O)(=O)c(cc2)ccc2-c(ccc3)cc3CN4C(C)CCC4C	520	3.284	79.11063	272.3177	5	0	1	3	0
274	C1CN(C)CCN1CCCc2c(F)cc(c(F)c2)S(=O)(=O)Nc(c3C)c(C)n(n3)C	548	3.261	55.99856	114.3982	3	0	1	2	0
275	C1CN(C)CCN1CCCc2cc(C)c(c(C)c2)S(=O)(=O)N(C)c(c3C)c(C)n(n3)C	850	3.071	65.69569	117.6582	2	0	0	2	0
276	CC(C)Cc1c(c(C)n(n1)C)NS(=O)(=O)c(cc2)ccc2CCCN3CCN(C)CC3	939	3.027	70.98451	166.3202	5	0	1	3	0
277	C1CN(C)CCN1CCCc2cc(C)c(c(C)c2)S(=O)(=O)Nc(c3C)c(C)n(n3)C	980	3.009	46.53551	135.7709	2	0	1	2	0

278	COC(=O)c1ccc(N2CCCCCC2)c(NS(=O)(=O)c2ccc(CCCCC3CCN(C)CC3)cc2)c1	0.34	6.469	115.1423	99.6496	8	0	1	5	3
279	O=S(=O)(Nc1c(N2CCCC2)ccc2ncccc12)c1ccc(Br)cc1	41	4.387	38.84964	193.6271	3	0	1	4	0
280	CN1CCC(CCCCc2ccc(S(=O)(=O)Nc3c(N4CCCC4)ccc4ncccc34)cc2)CC1	1.2	5.921	83.15506	180.7073	4	0	1	5	2
281	O=S(=O)(Nc1c(N2CCCCCC2)ccc2ncccc12)c1ccc(Br)cc1	36	4.444	45.4099	196.197	7	0	1	4	0
282	O=S(=O)(Nc1c(C2CCCC2)ccc2ncccc12)c1ccc(Br)cc1	64	4.194	52.11625	188.1228	5	0	1	4	0
283	CC(C)Cc1ccc2ncccc2c1NS(=O)(=O)c1ccc(CCCC2CCN(C)CC2)cc1	49	4.31	81.30529	170.6257	7	0	1	4	1
284	CC(C)Cc1ccc2ncccc2c1NS(=O)(=O)c1ccc(CCCCC2CCN(C)CC2)cc1	250	3.602	84.92027	176.342	7	0	1	4	2
285	CC(C)Cc1ccc2ncccc2c1NS(=O)(=O)c1ccc(CCCCC2CCNCC2)cc1	240	3.62	85.11712	176.6551	7	1	2	4	2
286	CC(C)Cc1ccc2ncccc2c1NS(=O)(=O)c1ccc(CCCN2CCN3CCC[C@@H]3C2)cc1	640	3.194	89.43194	185.9897	8	0	3	4	0
287	CC(C)Cc1ccc2ncccc2c1NS(=O)(=O)c1ccc(CCCCN2CCN3CCC[C@@H]3C2)cc1	3600	2.444	94.11743	185.3163	8	0	3	4	0
288	CC(C)Cc1ccc2ncccc2c1NS(=O)(=O)c1ccc(CCCN2CCCN(C)CC2)cc1	300	3.523	85.94592	165.5351	8	0	1	4	0
289	CC(C)Cc1ccc2ncccc2c1NS(=O)(=O)c1ccc(CCCCN2CCCN(C)CC2)cc1	1400	2.854	96.39272	176.0077	8	0	1	4	0

290	CC(C)Cc1ccc2ncccc2c1NS(=O)(=O)c1ccc(CCCN2CCN(C)CC2)cc1	550	3.26	80.73398	171.1926	8	0	1	4	0
291	CC(C)Cc1ccc2ncccc2c1NS(=O)(=O)c1ccc(CCCCN2CCNCC2)cc1	2000	2.699	78.26715	177.148	8	1	1	4	0
292	CC(C)Cc1ccc2ncccc2c1NS(=O)(=O)c1ccc(CCCCN2C[C@H](C)N[C@H](C)C2)cc1	10000	2	95.01874	175.9967	8	1	6	4	0
293	CC(C)Cc1ccc2ncccc2c1NS(=O)(=O)c1ccc(CCCO[C@H]2C[C@@H]3CC[C@H](C2)N3C)cc1	27000	1.569	84.90097	161.0079	7	0	1	4	2
294	CC(C)Cc1ccc2ncccc2c1NS(=O)(=O)c1ccc(CCCO[C@@H]2C[C@@H]3CC[C@H](C2)N3C)cc1	130	3.886	84.90097	161.0079	7	0	1	4	2
295	CC(C)Cc1ccc2ncccc2c1NS(=O)(=O)c1ccc(CCCO[C@@H]2CCCN(C)C2)cc1	1200	2.921	82.12256	177.0925	8	0	3	4	1
296	CC(C)Cc1ccc2ncccc2c1NS(=O)(=O)c1ccc(CCCOC[C@@H]2CNCCO2)cc1	390	3.409	79.38057	170.2496	8	1	1	4	2
297	CC(C)Cc1ccc2ncccc2c1NS(=O)(=O)c1ccc(CCCOC[C@@H]2CN(C)CCO2)cc1	340	3.469	89.06473	160.6168	8	0	1	4	2
298	CC(C)Cc1ccc2nn(C)cc2c1NS(=O)(=O)c1ccc(CCCC2CCN(C)CC2)cc1	280	3.553	91.84023	121.5931	6	0	1	3	1
299	CC(C)Cc1ccc2nn(C)cc2c1NS(=O)(=O)c1ccc(CCCCC2CCN(C)CC2)cc1	230	3.638	93.85416	121.3311	6	0	1	3	2
300	CC(C)Cc1ccc2nn(C)cc2c1NS(=O)(=O)c1ccc(CCCCN2CCN3CCC[C@@H]3C2)cc1	13000	1.886	102.1336	120.7908	7	0	3	3	0
301	CC(C)Cc1ccc2nn(C)cc2c1NS(=O)(=O)c1ccc(CCCN2CCCN(C)CC2)cc1	290	3.538	95.17438	121.7015	7	0	1	3	0

302	CC(C)Cc1ccc2nn(C)cc2c1NS(=O)(=O)c1ccc(CCCCN2CCCN(C)CC2)cc1	1200	2.921	102.9579	122.2612	7	0	1	3	0
303	CC(C)Cc1ccc2nn(C)cc2c1NS(=O)(=O)c1ccc(CCCOC[C@@H]2CCCN2C)cc1	2500	2.602	69.06892	118.4336	5	0	3	3	1
304	CC(C)Cc1ccc2nn(C)cc2c1NS(=O)(=O)c1ccc(CCCOC[C@H]2CCCN2C)cc1	730	3.137	69.06892	118.4336	5	0	3	3	1
305	CC(C)Cc1ccc2nn(C)cc2c1NS(=O)(=O)c1ccc(CCCO[C@H]2C[C@@H]3CC[C@H](C2)N3C)cc1	12000	1.921	92.84751	121.8948	6	0	1	3	2
306	CC(C)Cc1ccc2nn(C)cc2c1NS(=O)(=O)c1ccc(CCCOC[C@@H]2COCCN2C)cc1	9600	2.018	86.81116	121.3382	5	0	1	3	1
307	CC(C)Cc1ccc2nn(C)cc2c1NS(=O)(=O)c1ccc(CCCOC[C@H]2COCCN2C)cc1	4600	2.337	86.81116	121.3382	5	0	1	3	1
308	CC(C)Cc1ccc2nn(C)cc2c1NS(=O)(=O)c1ccc(CCCOC[C@@H]2CN(C)CCO2)cc1	190	3.721	93.95512	122.8373	7	0	1	3	2
309	CC(C)Cc1ccc2nn(C)cc2c1NS(=O)(=O)c1ccc(CCCOC[C@H]2CN(C)CCO2)cc1	130	3.886	93.95512	122.8373	7	0	1	3	2

Table S2: Details regarding performance of model 1

SN	Status	Exp. endpoint	Pred. by model eq.	Pred.Mod.Eq.Res.	Pred. LOO	Pred. LOO Res.	HAT i/i (h*=0.1161)	Std.Pred.Mod.Eq. Res.	Std.Pred.LOO Res.
1	Training	3	3.6497	0.6497	3.6712	0.6712	0.0319	0.7594	0.7845
2	Training	8.398	6.8548	-1.5432	6.8153	-1.5827	0.0249	-1.7973	-1.8432
3	Training	8.097	6.9591	-1.1379	6.9267	-1.1703	0.0276	-1.3271	-1.3648

4	Training	7.921	6.9776	-0.9434	6.9533	-0.9677	0.0252	-1.0988	-1.1272
5	Training	8.155	7.5437	-0.6113	7.5253	-0.6297	0.0292	-0.7135	-0.7349
6	Prediction	8.155	6.9005	-1.2545	-	-	0.0258	-1.4616	-1.4616
7	Training	7.432	7.1235	-0.3085	7.1141	-0.3179	0.0299	-0.3601	-0.3712
8	Prediction	8	6.6806	-1.3194	-	-	0.0736	-1.5765	-1.5765
9	Prediction	5.609	6.9689	1.3599	-	-	0.0898	1.6393	1.6393
10	Training	7.215	6.5601	-0.6549	6.4239	-0.7911	0.1721	-0.8278	-0.9999
11	Prediction	6.569	6.3819	-0.1871	-	-	0.1362	-0.2316	-0.2316
12	Prediction	5.821	7.6598	1.8388	-	-	0.0893	2.2159	2.2159
13	Training	8.155	7.1281	-1.0269	7.0961	-1.0589	0.0303	-1.1992	-1.2367
14	Prediction	8.097	7.3498	-0.7472	-	-	0.0361	-0.8752	-0.8752
15	Training	6.854	6.5735	-0.2805	6.5669	-0.2871	0.0229	-0.3264	-0.334
16	Training	7.229	6.9208	-0.3082	6.9125	-0.3165	0.0261	-0.3592	-0.3688
17	Training	6.377	6.9142	0.5372	6.9348	0.5578	0.037	0.6296	0.6537
18	Prediction	6.301	6.9002	0.5992	-	-	0.078	0.7176	0.7176
19	Prediction	7.208	7.6845	0.4765	-	-	0.0393	0.5591	0.5591
20	Training	8.699	7.7547	-0.9443	7.7145	-0.9845	0.0409	-1.1089	-1.1561
21	Training	7.77	6.8712	-0.8988	6.8341	-0.9359	0.0396	-1.0548	-1.0983
22	Prediction	7.77	7.0101	-0.7599	-	-	0.028	-0.8864	-0.8864
23	Prediction	5.77	6.9875	1.2175	-	-	0.0641	1.4473	1.4473
24	Training	8.301	7.4394	-0.8616	7.4048	-0.8962	0.0386	-1.0106	-1.0511
25	Training	6.921	6.2442	-0.6768	6.2284	-0.6926	0.0228	-0.7873	-0.8057
26	Prediction	6.456	6.2385	-0.2175	-	-	0.0229	-0.253	-0.253
27	Training	7.367	6.5224	-0.8446	6.4952	-0.8718	0.0311	-0.9868	-1.0185
28	Prediction	7.377	7.8031	0.4261	-	-	0.0395	0.5	0.5
29	Prediction	6.481	7.232	0.751	-	-	0.0857	0.9033	0.9033
30	Prediction	7.62	7.4077	-0.2123	-	-	0.032	-0.2481	-0.2481
31	Prediction	6.745	7.575	0.83	-	-	0.0345	0.9714	0.9714
32	Prediction	6.409	7.3295	0.9205	-	-	0.0485	1.0853	1.0853
33	Training	6.959	7.0809	0.1219	7.0886	0.1296	0.0589	0.1446	0.1536

34	Prediction	6.076	7.2054	1.1294	-	-	0.0582	1.3383	1.3383
35	Training	6.244	7.1336	0.8896	7.1874	0.9434	0.057	1.0536	1.1173
36	Prediction	5.699	3.7589	-1.9401	-	-	0.0166	-2.2499	-2.2499
37	Prediction	2.863	1.8568	-1.0062	-	-	0.1336	-1.2432	-1.2432
38	Prediction	2.824	3.7788	0.9548	-	-	0.0228	1.1108	1.1108
39	Training	2.77	3.7506	0.9806	3.7943	1.0243	0.0426	1.1525	1.2039
40	Training	2.678	3.8124	1.1344	3.8617	1.1837	0.0416	1.3327	1.3905
41	Prediction	2.638	3.8551	1.2171	-	-	0.0242	1.417	1.417
42	Training	2.638	3.2529	0.6149	3.2729	0.6349	0.0314	0.7186	0.7419
43	Prediction	4.77	4.7833	0.0133	-	-	0.0692	0.0159	0.0159
44	Training	5.569	5.4558	-0.1132	5.4473	-0.1217	0.0699	-0.135	-0.1451
45	Prediction	2.638	3.7092	1.0712	-	-	0.0532	1.266	1.266
46	Prediction	2.62	3.5407	0.9207	-	-	0.0382	1.0796	1.0796
47	Training	2.62	3.6736	1.0536	3.7332	1.1132	0.0535	1.2455	1.316
48	Prediction	2.585	2.7847	0.1997	-	-	0.0856	0.2401	0.2401
49	Training	2.538	3.6639	1.1259	3.705	1.167	0.0352	1.3183	1.3664
50	Prediction	2.474	2.022	-0.452	-	-	0.1049	-0.5494	-0.5494
51	Prediction	2.432	3.5928	1.1608	-	-	0.0415	1.3635	1.3635
52	Training	2.42	4.0183	1.5983	4.0815	1.6615	0.038	1.8741	1.9481
53	Prediction	2.398	4.8635	2.4655	-	-	0.0368	2.889	2.889
54	Training	2.367	2.7489	0.3819	2.7702	0.4032	0.0529	0.4513	0.4764
55	Prediction	2.349	3.7553	1.4063	-	-	0.0166	1.6309	1.6309
56	Prediction	2.215	3.1778	0.9628	-	-	0.0311	1.1248	1.1248
57	Training	2.201	3.5278	1.3268	3.5868	1.3858	0.0426	1.5594	1.6288
58	Prediction	2.032	3.5356	1.5036	-	-	0.0159	1.7431	1.7431
59	Training	5.086	6.344	1.258	6.3967	1.3107	0.0402	1.4767	1.5386
60	Prediction	4.796	6.6051	1.8091	-	-	0.0286	2.1109	2.1109
61	Prediction	4.131	6.0734	1.9424	-	-	0.0351	2.2742	2.2742
62	Training	6	6.1762	0.1762	6.1831	0.1831	0.0378	0.2066	0.2147
63	Training	5.62	6.6046	0.9846	6.6467	1.0267	0.041	1.1563	1.2058

64	Prediction	5.06	5.323	0.263	-	-	0.0539	0.3109	0.3109
65	Training	5.796	6.7879	0.9919	6.8345	1.0385	0.0448	1.1672	1.2219
66	Prediction	6.959	7.0881	0.1291	-	-	0.048	0.1522	0.1522
67	Prediction	5.377	6.9609	1.5839	-	-	0.0269	1.8466	1.8466
68	Prediction	4.347	6.6868	2.3398	-	-	0.0238	2.7235	2.7235
69	Training	6.125	6.7495	0.6245	6.7642	0.6392	0.023	0.7266	0.7437
70	Training	5.367	6.4456	1.0786	6.4738	1.1068	0.0255	1.2565	1.2894
71	Prediction	8.155	6.7719	-1.3831	-	-	0.0264	-1.612	-1.612
72	Prediction	6.921	6.6122	-0.3088	-	-	0.0239	-0.3594	-0.3594
73	Prediction	8.523	6.9532	-1.5698	-	-	0.0295	-1.8325	-1.8325
74	Prediction	8.046	6.6472	-1.3988	-	-	0.0272	-1.631	-1.631
75	Prediction	7.921	6.5698	-1.3512	-	-	0.0273	-1.5756	-1.5756
76	Prediction	7.229	6.2914	-0.9376	-	-	0.0266	-1.0928	-1.0928
77	Prediction	7.602	7.9293	0.3273	-	-	0.0404	0.3843	0.3843
78	Prediction	8.398	7.9841	-0.4139	-	-	0.0412	-0.4861	-0.4861
79	Training	7.77	6.7438	-1.0262	6.7195	-1.0505	0.0231	-1.194	-1.2223
80	Training	1.752	2.235	0.483	2.281	0.529	0.087	0.5813	0.6367
81	Prediction	1.699	2.2563	0.5573	-	-	0.0861	0.6704	0.6704
82	Prediction	1.155	2.6023	1.4473	-	-	0.0907	1.7455	1.7455
83	Training	1.15	2.0132	0.8632	2.1173	0.9673	0.1076	1.0508	1.1776
84	Prediction	1.06	2.0185	0.9585	-	-	0.0986	1.1611	1.1611
85	Prediction	7.638	6.7159	-0.9221	-	-	0.0267	-1.0749	-1.0749
86	Training	7.602	6.4658	-1.1362	6.4329	-1.1691	0.0282	-1.3255	-1.3639
87	Training	4.77	4.5057	-0.2643	4.4841	-0.2859	0.0755	-0.3161	-0.3419
88	Training	5.337	6.3391	1.0021	6.3751	1.0381	0.0347	1.1729	1.2151
89	Training	6.409	6.4576	0.0486	6.4588	0.0498	0.0223	0.0566	0.0579
90	Training	5.456	6.5882	1.1322	6.6277	1.1717	0.0337	1.3246	1.3708
91	Prediction	7.481	6.7499	-0.7311	-	-	0.0259	-0.852	-0.852
92	Prediction	6.721	6.8539	0.1329	-	-	0.0295	0.1552	0.1552
93	Training	8.097	6.9071	-1.1899	6.8715	-1.2255	0.0291	-1.3887	-1.4303

94	Prediction	6.886	7.0377	0.1517	-	-	0.0323	0.1773	0.1773
95	Training	8.398	6.9121	-1.4859	6.8704	-1.5276	0.0273	-1.7327	-1.7813
96	Training	7.398	7.1894	-0.2086	7.1824	-0.2156	0.0323	-0.2439	-0.252
97	Prediction	8.301	7.2322	-1.0688	-	-	0.0333	-1.2502	-1.2502
98	Training	7.538	7.3118	-0.2262	7.3034	-0.2346	0.0357	-0.2649	-0.2747
99	Prediction	5.137	3.4053	-1.7317	-	-	0.0726	-2.0679	-2.0679
100	Prediction	5.446	4.1509	-1.2951	-	-	0.0593	-1.5357	-1.5357
101	Prediction	8.398	7.4565	-0.9415	-	-	0.036	-1.1028	-1.1028
102	Training	7.658	7.5853	-0.0727	7.5826	-0.0754	0.0357	-0.0852	-0.0884
103	Training	8.046	6.9046	-1.1414	6.8566	-1.1894	0.0404	-1.34	-1.3964
104	Prediction	7.77	7.561	-0.209	-	-	0.0454	-0.246	-0.246
105	Training	6.721	7.1587	0.4377	7.1716	0.4506	0.0286	0.5108	0.5258
106	Training	6.432	7.5924	1.1604	7.6339	1.2019	0.0346	1.3582	1.4068
107	Prediction	6.328	6.5271	0.1991	-	-	0.0276	0.2322	0.2322
108	Training	6.523	6.9558	0.4328	6.9685	0.4455	0.0286	0.5049	0.5198
109	Prediction	7.745	7.5052	-0.2398	-	-	0.0349	-0.2808	-0.2808
110	Prediction	6.409	7.4799	1.0709	-	-	0.0356	1.254	1.254
111	Training	0.971	2.1803	1.2093	2.2929	1.3219	0.0852	1.4541	1.5894
112	Training	5.398	4.2155	-1.1825	4.149	-1.249	0.0532	-1.3976	-1.4762
113	Prediction	5.398	4.107	-1.291	-	-	0.0374	-1.5133	-1.5133
114	Training	5.301	3.9976	-1.3034	3.9743	-1.3267	0.0176	-1.5123	-1.5394
115	Prediction	5.301	4.3524	-0.9486	-	-	0.0275	-1.1063	-1.1063
116	Prediction	5.523	4.8687	-0.6543	-	-	0.0558	-0.7743	-0.7743
117	Prediction	5.222	4.687	-0.535	-	-	0.0274	-0.6239	-0.6239
118	Training	7.62	7.8632	0.2432	7.8737	0.2537	0.0414	0.2857	0.2981
119	Training	7.921	7.2648	-0.6562	7.2421	-0.6789	0.0334	-0.7675	-0.7941
120	Training	6.745	7.7719	1.0269	7.8212	1.0762	0.0458	1.209	1.267
121	Training	6.602	7.5013	0.8993	7.5409	0.9389	0.0422	1.0567	1.1032
122	Prediction	8.046	7.3473	-0.6987	-	-	0.0328	-0.817	-0.817
123	Training	6.796	6.8677	0.0717	6.8696	0.0736	0.0254	0.0835	0.0857

124	Training	8.398	7.8535	-0.5445	7.8291	-0.5689	0.0428	-0.64	-0.6687
125	Training	8.301	7.7069	-0.5941	7.6818	-0.6192	0.0405	-0.6976	-0.727
126	Prediction	8.301	7.4839	-0.8171	-	-	0.0358	-0.957	-0.957
127	Prediction	8.097	7.1393	-0.9577	-	-	0.0282	-1.1173	-1.1173
128	Training	7.131	7.1674	0.0364	7.1693	0.0383	0.0494	0.0429	0.0452
129	Training	6.553	7.2332	0.6802	7.2647	0.7117	0.0443	0.8002	0.8373
130	Prediction	7.347	7.1778	-0.1692	-	-	0.0498	-0.1997	-0.1997
131	Prediction	6.824	7.1546	0.3306	-	-	0.0414	0.3883	0.3883
132	Training	7.328	7.0363	-0.2917	7.0243	-0.3037	0.0397	-0.3423	-0.3564
133	Prediction	5.097	3.8736	-1.2234	-	-	0.0663	-1.456	-1.456
134	Training	5.097	4.3656	-0.7314	4.345	-0.752	0.0275	-0.8529	-0.877
135	Prediction	5.046	4.2245	-0.8215	-	-	0.0502	-0.9694	-0.9694
136	Prediction	3.252	3.8499	0.5979	-	-	0.0126	0.692	0.692
137	Training	5.046	4.56	-0.486	4.5507	-0.4953	0.0187	-0.5642	-0.575
138	Prediction	5	3.7305	-1.2695	-	-	0.0381	-1.4886	-1.4886
139	Training	5	3.5392	-1.4608	3.4484	-1.5516	0.0586	-1.7314	-1.8391
140	Prediction	5	3.9818	-1.0182	-	-	0.0114	-1.1777	-1.1777
141	Training	4.959	3.8419	-1.1171	3.791	-1.168	0.0436	-1.3136	-1.3734
142	Prediction	4.959	4.0361	-0.9229	-	-	0.0115	-1.0675	-1.0675
143	Training	4.959	4.3416	-0.6174	4.3244	-0.6346	0.0271	-0.7199	-0.7399
144	Prediction	4.921	4.7992	-0.1218	-	-	0.0378	-0.1428	-0.1428
145	Training	4.886	4.1827	-0.7033	4.1738	-0.7122	0.0125	-0.8139	-0.8242
146	Training	4.854	3.9887	-0.8653	3.9776	-0.8764	0.0127	-1.0015	-1.0143
147	Training	4.796	3.6936	-1.1024	3.6594	-1.1366	0.0301	-1.2873	-1.3272
148	Prediction	4.77	3.2655	-1.5045	-	-	0.0473	-1.7726	-1.7726
149	Training	3.208	2.2272	-0.9808	1.9899	-1.2181	0.1949	-1.257	-1.5613
150	Training	4.77	4.095	-0.675	4.0836	-0.6864	0.0167	-0.7828	-0.7961
151	Prediction	4.745	3.4618	-1.2832	-	-	0.0349	-1.5021	-1.5021
152	Training	4.745	4.7161	-0.0289	4.7145	-0.0305	0.0529	-0.0342	-0.0361
153	Prediction	4.721	3.7737	-0.9473	-	-	0.0183	-1.0995	-1.0995

154	Training	4.721	5.4033	0.6823	5.443	0.722	0.055	0.8072	0.8542
155	Prediction	3.201	4.3547	1.1537	-	-	0.015	1.3369	1.3369
156	Prediction	4.721	4.945	0.224	-	-	0.0353	0.2623	0.2623
157	Training	4.721	3.6885	-1.0325	3.6667	-1.0543	0.0207	-1.1999	-1.2252
158	Prediction	6.523	6.8897	0.3667	-	-	0.044	0.4313	0.4313
159	Training	6.523	6.321	-0.202	6.3124	-0.2106	0.0411	-0.2372	-0.2474
160	Prediction	7.046	6.7323	-0.3137	-	-	0.0525	-0.3706	-0.3706
161	Training	8	6.3364	-1.6636	6.2568	-1.7432	0.0457	-1.9584	-2.0522
162	Prediction	7.222	6.5025	-0.7195	-	-	0.0489	-0.8484	-0.8484
163	Training	7.523	6.5203	-1.0027	6.4744	-1.0486	0.0438	-1.1792	-1.2332
164	Training	7.097	7.4085	0.3115	7.4221	0.3251	0.0419	0.366	0.382
165	Training	5.745	7.8054	2.0604	7.8967	2.1517	0.0424	2.4214	2.5288
166	Training	7	5.9552	-1.0448	5.9066	-1.0934	0.0444	-1.2292	-1.2864
167	Training	5.187	5.7249	0.5379	5.7445	0.5575	0.0351	0.6297	0.6527
168	Training	5.886	6.947	1.061	6.9887	1.1027	0.0378	1.2439	1.2928
169	Prediction	6.699	6.77	0.071	-	-	0.0352	0.0831	0.0831
170	Training	6.301	6.4274	0.1264	6.4324	0.1314	0.0381	0.1482	0.1541
171	Prediction	6.155	6.8467	0.6917	-	-	0.037	0.8106	0.8106
172	Prediction	5.357	5.6563	0.2993	-	-	0.0263	0.3488	0.3488
173	Prediction	7.097	6.6161	-0.4809	-	-	0.0311	-0.5619	-0.5619
174	Prediction	7.523	6.7085	-0.8145	-	-	0.0314	-0.9518	-0.9518
175	Prediction	4.824	6.1917	1.3677	-	-	0.0301	1.5972	1.5972
176	Prediction	4.854	5.6154	0.7614	-	-	0.0351	0.8914	0.8914
177	Training	4.699	3.9776	-0.7214	3.9692	-0.7298	0.0116	-0.8344	-0.8442
178	Training	4.678	5.0266	0.3486	5.0408	0.3628	0.0392	0.409	0.4257
179	Prediction	4.678	3.6848	-0.9932	-	-	0.0392	-1.1652	-1.1652
180	Training	4.678	3.7174	-0.9606	3.7	-0.978	0.0178	-1.1147	-1.1349
181	Prediction	4.678	3.8146	-0.8634	-	-	0.0231	-1.0046	-1.0046
182	Prediction	3.187	3.6716	0.4846	-	-	0.0471	0.5709	0.5709
183	Prediction	4.658	3.8593	-0.7987	-	-	0.0176	-0.9268	-0.9268

184	Training	4.602	4.8578	0.2558	4.8661	0.2641	0.0312	0.2989	0.3085
185	Prediction	4.538	3.9749	-0.5631	-	-	0.0126	-0.6517	-0.6517
186	Training	4.523	4.0163	-0.5067	4.0097	-0.5133	0.0129	-0.5865	-0.5942
187	Training	4.481	3.8734	-0.6076	3.8654	-0.6156	0.013	-0.7033	-0.7126
188	Prediction	4.481	4.346	-0.135	-	-	0.0501	-0.1593	-0.1593
189	Prediction	4.469	3.9698	-0.4992	-	-	0.0457	-0.5877	-0.5877
190	Prediction	4.444	3.3798	-1.0642	-	-	0.0246	-1.2392	-1.2392
191	Training	4.432	4.7657	0.3337	4.7753	0.3433	0.0281	0.3892	0.4005
192	Prediction	4.409	3.4993	-0.9097	-	-	0.0276	-1.0609	-1.0609
193	Training	4.398	3.6137	-0.7843	3.472	-0.926	0.1531	-0.9801	-1.1572
194	Training	4.387	4.0497	-0.3373	4.0452	-0.3418	0.0129	-0.3905	-0.3956
195	Training	4.337	3.9547	-0.3823	3.9345	-0.4025	0.0501	-0.4511	-0.4749
196	Prediction	4.328	4.3187	-0.0093	-	-	0.0138	-0.0107	-0.0107
197	Training	4.328	4.835	0.507	4.8475	0.5195	0.0241	0.5902	0.6048
198	Prediction	4.328	3.4937	-0.8343	-	-	0.0284	-0.9733	-0.9733
199	Training	4.292	3.9476	-0.3444	3.9289	-0.3631	0.0513	-0.4067	-0.4287
200	Prediction	4.292	4.0832	-0.2088	-	-	0.0123	-0.2416	-0.2416
201	Prediction	4.284	3.4822	-0.8018	-	-	0.0188	-0.9309	-0.9309
202	Training	4.268	4.8416	0.5736	4.8596	0.5916	0.0304	0.6699	0.6909
203	Training	3.131	2.9299	-0.2011	2.9248	-0.2062	0.0247	-0.2342	-0.2402
204	Prediction	4.252	4.4264	0.1744	-	-	0.05	0.2058	0.2058
205	Training	4.244	4.2432	-0.0008	4.2432	-0.0008	0.0132	-0.0009	-0.0009
206	Prediction	4.237	3.9318	-0.3052	-	-	0.0124	-0.3532	-0.3532
207	Training	4.222	4.0581	-0.1639	4.0491	-0.1729	0.0522	-0.1936	-0.2042
208	Prediction	4.215	4.4603	0.2453	-	-	0.0135	0.284	0.284
209	Prediction	4.194	3.8367	-0.3573	-	-	0.0231	-0.4157	-0.4157
210	Prediction	3.131	4.0685	0.9375	-	-	0.0132	1.0854	1.0854
211	Training	4.149	4.4887	0.3397	4.5137	0.3647	0.0685	0.4048	0.4345
212	Training	4.125	4.3647	0.2397	4.3691	0.2441	0.0181	0.2782	0.2833
213	Training	4.119	4.1729	0.0539	4.1735	0.0545	0.012	0.0623	0.0631

214	Prediction	4.119	3.1992	-0.9198	-	-	0.03	-1.074	-1.074
215	Training	5.004	5.6238	0.6198	5.6449	0.6409	0.0329	0.7248	0.7494
216	Training	6.046	5.7323	-0.3137	5.7198	-0.3262	0.0383	-0.3679	-0.3825
217	Prediction	6.523	6.7624	0.2394	-	-	0.0495	0.2824	0.2824
218	Training	5.42	6.7125	1.2925	6.8536	1.4336	0.0984	1.5654	1.7363
219	Prediction	6	6.5988	0.5988	-	-	0.0338	0.7006	0.7006
220	Prediction	7.398	7.3551	-0.0429	-	-	0.0377	-0.0503	-0.0503
221	Training	7.398	7.2189	-0.1791	7.2104	-0.1876	0.0452	-0.2108	-0.2208
222	Training	6.699	6.8675	0.1685	6.8785	0.1795	0.0611	0.2	0.213
223	Prediction	7.398	6.9148	-0.4832	-	-	0.053	-0.571	-0.571
224	Prediction	6.745	7.4713	0.7263	-	-	0.0571	0.8602	0.8602
225	Prediction	4.114	3.9814	-0.1326	-	-	0.0174	-0.1538	-0.1538
226	Training	4.097	4.5067	0.4097	4.5262	0.4292	0.0453	0.4822	0.5051
227	Prediction	4.092	4.1913	0.0993	-	-	0.0492	0.1171	0.1171
228	Training	4.056	3.881	-0.175	3.8758	-0.1802	0.0287	-0.2042	-0.2102
229	Prediction	3.119	3.0482	-0.0708	-	-	0.0348	-0.0828	-0.0828
230	Prediction	4.027	3.9493	-0.0777	-	-	0.0127	-0.09	-0.09
231	Training	4.018	4.9064	0.8884	4.9271	0.9091	0.0227	1.0335	1.0575
232	Prediction	4	4.7154	0.7154	-	-	0.0235	0.8326	0.8326
233	Training	3.996	3.9438	-0.0522	3.9413	-0.0547	0.0447	-0.0615	-0.0643
234	Training	3.959	4.0011	0.0421	4.0034	0.0444	0.0523	0.0497	0.0525
235	Prediction	3.959	5.0083	1.0493	-	-	0.0108	1.2133	1.2133
236	Training	3.921	3.6794	-0.2416	3.6757	-0.2453	0.0152	-0.28	-0.2843
237	Prediction	3.921	4.4465	0.5255	-	-	0.015	0.609	0.609
238	Prediction	3.873	4.2909	0.4179	-	-	0.0557	0.4946	0.4946
239	Training	3.854	3.5366	-0.3174	3.5316	-0.3224	0.0156	-0.3679	-0.3737
240	Prediction	3.851	2.9158	-0.9352	-	-	0.1121	-1.1414	-1.1414
241	Training	3.842	4.6071	0.7651	4.623	0.781	0.0205	0.889	0.9075
242	Prediction	3.83	4.3104	0.4804	-	-	0.0512	0.5671	0.5671
243	Prediction	3.824	3.5384	-0.2856	-	-	0.0139	-0.3308	-0.3308

244	Prediction	3.114	4.8727	1.7587	-	-	0.029	2.0525	2.0525
245	Training	3.824	2.9451	-0.8789	2.9054	-0.9186	0.0432	-1.0333	-1.08
246	Training	3.815	3.8436	0.0286	3.8441	0.0291	0.0163	0.0331	0.0337
247	Prediction	3.785	4.0231	0.2381	-	-	0.0293	0.2779	0.2779
248	Training	3.775	3.7378	-0.0372	3.7372	-0.0378	0.015	-0.0432	-0.0438
249	Training	3.114	4.0361	0.9221	4.0706	0.9566	0.0361	1.0801	1.1206
250	Prediction	3.762	3.8549	0.0929	-	-	0.0162	0.1078	0.1078
251	Training	3.759	4.209	0.45	4.2139	0.4549	0.0107	0.5203	0.5259
252	Training	3.745	3.0295	-0.7155	3.0126	-0.7324	0.023	-0.8325	-0.8521
253	Prediction	3.745	4.0218	0.2768	-	-	0.024	0.3222	0.3222
254	Training	3.721	5.059	1.338	5.1003	1.3793	0.0299	1.5623	1.6105
255	Prediction	3.699	2.6296	-1.0694	-	-	0.2383	-1.4092	-1.4092
256	Prediction	3.678	3.7101	0.0321	-	-	0.0443	0.0377	0.0377
257	Training	3.678	3.6414	-0.0366	3.6399	-0.0381	0.0387	-0.0429	-0.0446
258	Training	3.658	3.204	-0.454	3.1964	-0.4616	0.0164	-0.5265	-0.5352
259	Training	3.633	4.4511	0.8181	4.4625	0.8295	0.0137	0.9474	0.9605
260	Prediction	3.631	4.082	0.451	-	-	0.024	0.525	0.525
261	Training	3.62	4.1789	0.5589	4.189	0.569	0.0177	0.6485	0.6602
262	Prediction	3.62	3.0339	-0.5861	-	-	0.1107	-0.7147	-0.7147
263	Prediction	3.602	4.0863	0.4843	-	-	0.0134	0.5608	0.5608
264	Training	3.602	3.0721	-0.5299	3.0517	-0.5503	0.0371	-0.6211	-0.645
265	Training	3.565	2.6355	-0.9295	2.5655	-0.9995	0.07	-1.1085	-1.1919
266	Training	3.509	4.1421	0.6331	4.1593	0.6503	0.0265	0.7379	0.7579
267	Prediction	3.503	4.0027	0.4997	-	-	0.0123	0.5783	0.5783
268	Prediction	3.481	3.2668	-0.2142	-	-	0.0159	-0.2484	-0.2484
269	Training	3.481	4.9565	1.4755	5.0001	1.5191	0.0287	1.7217	1.7726
270	Training	3.092	4.1273	1.0353	4.1439	1.0519	0.0157	1.2001	1.2193
271	Prediction	3.432	3.935	0.503	-	-	0.0255	0.586	0.586
272	Prediction	3.398	3.6455	0.2475	-	-	0.018	0.2872	0.2872
273	Training	3.284	4.5188	1.2348	4.5377	1.2537	0.015	1.4309	1.4527

274	Prediction	3.261	2.813	-0.448	-	-	0.0261	-0.522	-0.522
275	Prediction	3.071	3.4986	0.4276	-	-	0.0357	0.5007	0.5007
276	Training	3.027	3.3917	0.3647	3.3973	0.3703	0.0151	0.4226	0.4291
277	Prediction	3.009	2.8686	-0.1404	-	-	0.0344	-0.1643	-0.1643
278	Training	6.469	3.6497	-2.8193	3.4737	-2.9953	0.0588	-3.3419	-3.5505
279	Prediction	4.387	2.9983	-1.3887	-	-	0.0302	-1.6217	-1.6217
280	Prediction	5.921	4.0003	-1.9207	-	-	0.0187	-2.2298	-2.2298
281	Training	4.444	2.6359	-1.8081	2.5599	-1.8841	0.0404	-2.1226	-2.2119
282	Training	4.194	3.0423	-1.1517	3.0181	-1.1759	0.0206	-1.3384	-1.3665
283	Training	4.31	3.4353	-0.8747	3.4131	-0.8969	0.0248	-1.0186	-1.0445
284	Prediction	3.602	3.586	-0.016	-	-	0.0258	-0.0186	-0.0186
285	Training	3.62	3.9072	0.2872	3.9236	0.3036	0.054	0.3396	0.3589
286	Training	3.194	3.1708	-0.0232	3.1694	-0.0246	0.0567	-0.0275	-0.0291
287	Training	2.444	3.2977	0.8537	3.3518	0.9078	0.0596	1.0124	1.0766
288	Prediction	3.523	3.3811	-0.1419	-	-	0.0345	-0.1661	-0.1661
289	Training	2.854	3.7654	0.9114	3.8036	0.9496	0.0403	1.0699	1.1148
290	Training	3.26	3.2815	0.0215	3.2822	0.0222	0.0334	0.0252	0.026
291	Prediction	2.699	3.8161	1.1171	-	-	0.0615	1.3261	1.3261
292	Prediction	2	3.0751	1.0751	-	-	0.1915	1.3751	1.3751
293	Training	1.569	3.4557	1.8867	3.506	1.937	0.026	2.1985	2.2571
294	Training	3.886	3.4557	-0.4303	3.4442	-0.4418	0.026	-0.5014	-0.5148
295	Training	2.921	2.8886	-0.0324	2.8868	-0.0342	0.0538	-0.0383	-0.0405
296	Training	3.409	3.7892	0.3802	3.8144	0.4054	0.062	0.4515	0.4814
297	Training	3.469	3.4277	-0.0413	3.4262	-0.0428	0.0357	-0.0483	-0.0501
298	Prediction	3.553	3.4609	-0.0921	-	-	0.0259	-0.1073	-0.1073
299	Prediction	3.638	3.5157	-0.1223	-	-	0.0273	-0.1426	-0.1426
300	Prediction	1.886	3.1209	1.2349	-	-	0.059	1.464	1.464
301	Training	3.538	3.4137	-0.1243	3.4096	-0.1284	0.032	-0.1453	-0.1501
302	Prediction	2.921	3.6387	0.7177	-	-	0.0387	0.8419	0.8419
303	Training	2.602	2.4501	-0.1519	2.4438	-0.1582	0.0397	-0.1783	-0.1857

304	Training	3.137	2.4501	-0.6869	2.4217	-0.7153	0.0397	-0.8061	-0.8394
305	Training	1.921	3.492	1.571	3.5349	1.6139	0.0266	1.8312	1.8812
306	Training	2.018	3.4589	1.4409	3.491	1.473	0.0218	1.6754	1.7129
307	Training	2.337	3.4589	1.1219	3.4839	1.1469	0.0218	1.3045	1.3336
308	Prediction	3.721	3.3888	-0.3322	-	-	0.0312	-0.3881	-0.3881
309	Prediction	3.886	3.3888	-0.4972	-	-	0.0312	-0.5809	-0.5809

Table S3: Details regarding performance of model 2

SN	Status	Exp. endpoint	Pred. by model eq.	Pred.Mod.Eq.Res.	Pred. LOO	Pred. LOO Res.	HAT i/i (h*=0.1169)	Std.Pred.Mod.Eq. Res.	Std.Pred.LOO Res.
1	Prediction	3	3.9731	0.9731	-	-	0.0371	1.1073	1.1073
2	Prediction	8.398	6.9793	-1.4187	-	-	0.0261	-1.6052	-1.6052
3	Prediction	8.097	7.0055	-1.0915	-	-	0.0293	-1.2371	-1.2371
4	Prediction	7.921	7.1059	-0.8151	-	-	0.0243	-0.9215	-0.9215
5	Prediction	8.155	7.606	-0.549	-	-	0.0279	-0.6218	-0.6218
6	Training	8.155	7.0185	-1.1365	6.9869	-1.1681	0.0271	-1.2866	-1.3225
7	Prediction	7.432	7.2973	-0.1347	-	-	0.0287	-0.1527	-0.1527
8	Training	8	6.5525	-1.4475	6.4524	-1.5476	0.0647	-1.6713	-1.7869
9	Training	5.609	6.6729	1.0639	6.7557	1.1467	0.0723	1.2334	1.3294
10	Prediction	7.215	6.3034	-0.9116	-	-	0.1567	-1.1084	-1.1084
11	Training	6.569	5.9053	-0.6637	5.8339	-0.7351	0.0972	-0.78	-0.864
12	Training	5.821	7.4179	1.5969	7.5495	1.7285	0.0761	1.8552	2.0081
13	Prediction	8.155	6.9772	-1.1778	-	-	0.0355	-1.3391	-1.3391
14	Training	8.097	7.3844	-0.7126	7.3596	-0.7374	0.0337	-0.8095	-0.8377

15	Prediction	6.854	6.4776	-0.3764	-	-	0.0249	-0.4257	-0.4257
16	Prediction	7.229	6.7874	-0.4416	-	-	0.0293	-0.5005	-0.5005
17	Prediction	6.377	6.5943	0.2173	-	-	0.0335	0.2468	0.2468
18	Training	6.301	6.3916	0.0906	6.3974	0.0964	0.0609	0.1044	0.1111
19	Training	7.208	7.5314	0.3234	7.5473	0.3393	0.0469	0.3699	0.3881
20	Prediction	8.699	7.5946	-1.1044	-	-	0.0489	-1.2645	-1.2645
21	Prediction	7.77	6.6619	-1.1081	-	-	0.0503	-1.2698	-1.2698
22	Training	7.77	7.1935	-0.5765	7.1775	-0.5925	0.027	-0.6526	-0.6707
23	Training	5.77	6.5276	0.7576	6.5684	0.7984	0.0512	0.8684	0.9152
24	Prediction	8.301	7.1227	-1.1783	-	-	0.0377	-1.3413	-1.3413
25	Prediction	6.921	6.059	-0.862	-	-	0.0217	-0.9731	-0.9731
26	Training	6.456	6.0534	-0.4026	6.0445	-0.4115	0.0217	-0.4545	-0.4646
27	Prediction	7.367	6.2867	-1.0803	-	-	0.0379	-1.2298	-1.2298
28	Training	7.377	7.5737	0.1967	7.583	0.206	0.0453	0.2248	0.2354
29	Training	6.481	6.6858	0.2048	6.7003	0.2193	0.0665	0.2366	0.2535
30	Training	7.62	7.2194	-0.4006	7.2043	-0.4157	0.0362	-0.4557	-0.4728
31	Training	6.745	7.3679	0.6229	7.3932	0.6482	0.039	0.7095	0.7383
32	Training	6.409	7.0717	0.6627	7.1147	0.7057	0.0608	0.7636	0.8131
33	Prediction	6.959	7.1194	0.1604	-	-	0.055	0.1842	0.1842
34	Training	6.076	7.1067	1.0307	7.1594	1.0834	0.0486	1.1799	1.2403
35	Prediction	6.244	7.0431	0.7991	-	-	0.0477	0.9144	0.9144
36	Training	5.699	3.669	-2.03	3.6371	-2.0619	0.0155	-2.2845	-2.3205
37	Training	2.863	2.3957	-0.4673	2.3453	-0.5177	0.0974	-0.5492	-0.6085
38	Training	2.824	3.8326	1.0086	3.8573	1.0333	0.0239	1.14	1.1679
39	Prediction	2.77	4.0259	1.2559	-	-	0.0484	1.4376	1.4376
40	Prediction	2.678	4.0598	1.3818	-	-	0.0409	1.5755	1.5755
41	Training	2.638	3.9402	1.3022	3.9666	1.3286	0.0199	1.4688	1.4986
42	Prediction	2.638	3.4569	0.8189	-	-	0.0243	0.9258	0.9258
43	Training	4.77	4.641	-0.129	4.6312	-0.1388	0.0705	-0.1494	-0.1607
44	Prediction	5.569	5.4446	-0.1244	-	-	0.0756	-0.1445	-0.1445

45	Training	2.638	3.3622	0.7242	3.3936	0.7556	0.0415	0.826	0.8618
46	Training	2.62	3.812	1.192	3.8537	1.2337	0.0337	1.3541	1.4014
47	Prediction	2.62	3.6785	1.0585	-	-	0.05	1.2126	1.2126
48	Training	2.585	2.7411	0.1561	2.7562	0.1712	0.088	0.1825	0.2001
49	Prediction	2.538	3.761	1.223	-	-	0.0293	1.3861	1.3861
50	Training	2.474	2.4731	-0.0009	2.4731	-0.0009	0.0794	-0.001	-0.0011
51	Training	2.432	3.4709	1.0389	3.5139	1.0819	0.0398	1.1839	1.2329
52	Prediction	2.42	3.8224	1.4024	-	-	0.0402	1.5985	1.5985
53	Training	2.398	4.5065	2.1085	4.5675	2.1695	0.0281	2.3883	2.4573
54	Prediction	2.367	2.7944	0.4274	-	-	0.0544	0.4908	0.4908
55	Training	2.349	3.6658	1.3168	3.6865	1.3375	0.0155	1.4819	1.5052
56	Training	2.215	3.3415	1.1265	3.3705	1.1555	0.0251	1.274	1.3068
57	Prediction	2.201	3.7736	1.5726	-	-	0.0429	1.795	1.795
58	Training	2.032	3.5875	1.5555	3.6092	1.5772	0.0138	1.749	1.7734
59	Prediction	5.086	6.1705	1.0845	-	-	0.0463	1.2401	1.2401
60	Training	4.796	6.6838	1.8878	6.7395	1.9435	0.0286	2.1388	2.2019
61	Training	4.131	5.8065	1.6755	5.8698	1.7388	0.0364	1.906	1.978
62	Prediction	6	5.9579	-0.0421	-	-	0.0415	-0.048	-0.048
63	Prediction	5.62	6.4027	0.7827	-	-	0.0477	0.8956	0.8956
64	Training	5.06	5.2537	0.1937	5.2654	0.2054	0.0569	0.2228	0.2362
65	Prediction	5.796	6.7785	0.9825	-	-	0.0417	1.1207	1.1207
66	Training	6.959	7.05	0.091	7.0541	0.0951	0.0433	0.1039	0.1086
67	Training	5.377	6.7376	1.3606	6.7796	1.4026	0.03	1.5426	1.5902
68	Training	4.347	6.4953	2.1483	6.554	2.207	0.0266	2.4314	2.4979
69	Prediction	6.125	6.8902	0.7652	-	-	0.0236	0.8648	0.8648
70	Prediction	5.367	6.5547	1.1877	-	-	0.0238	1.3423	1.3423
71	Training	8.155	6.5672	-1.5878	6.52	-1.635	0.0289	-1.7992	-1.8527
72	Training	6.921	6.4276	-0.4934	6.4142	-0.5068	0.0264	-0.5584	-0.5735
73	Training	8.523	7.1471	-1.3759	7.1072	-1.4158	0.0282	-1.5585	-1.6037
74	Training	8.046	6.8724	-1.1736	6.8413	-1.2047	0.0258	-1.3278	-1.363

75	Training	7.921	6.7922	-1.1288	6.7608	-1.1602	0.0271	-1.2779	-1.3135
76	Training	7.229	6.5534	-0.6756	6.536	-0.693	0.0251	-0.7641	-0.7838
77	Training	7.602	7.9569	0.3549	7.971	0.369	0.0383	0.404	0.4201
78	Training	8.398	8.0054	-0.3926	7.9895	-0.4085	0.039	-0.4472	-0.4653
79	Prediction	7.77	6.8848	-0.8852	-	-	0.0238	-1.0004	-1.0004
80	Prediction	1.752	2.4638	0.7118	-	-	0.0772	0.8274	0.8274
81	Training	1.699	2.6893	0.9903	2.756	1.057	0.0631	1.1424	1.2194
82	Training	1.155	2.9858	1.8308	3.1234	1.9684	0.0699	2.1197	2.279
83	Prediction	1.15	2.4638	1.3138	-	-	0.082	1.5311	1.5311
84	Training	1.06	2.4029	1.3429	2.5174	1.4574	0.0786	1.5621	1.6954
85	Training	7.638	6.791	-0.847	6.7691	-0.8689	0.0251	-0.958	-0.9826
86	Prediction	7.602	6.3562	-1.2458	-	-	0.0311	-1.4133	-1.4133
87	Prediction	4.77	4.5877	-0.1823	-	-	0.0746	-0.2116	-0.2116
88	Prediction	5.337	6.3053	0.9683	-	-	0.0373	1.1019	1.1019
89	Prediction	6.409	6.2913	-0.1177	-	-	0.0246	-0.1331	-0.1331
90	Prediction	5.456	6.2744	0.8184	-	-	0.0306	0.9281	0.9281
91	Training	7.481	6.6171	-0.8639	6.5894	-0.8916	0.031	-0.98	-1.0114
92	Training	6.721	6.5758	-0.1452	6.5714	-0.1496	0.0294	-0.1646	-0.1696
93	Prediction	8.097	6.9672	-1.1298	-	-	0.0264	-1.2785	-1.2785
94	Training	6.886	6.95	0.064	6.9518	0.0658	0.0267	0.0724	0.0744
95	Prediction	8.398	6.7612	-1.6368	-	-	0.0329	-1.8585	-1.8585
96	Prediction	7.398	6.8759	-0.5221	-	-	0.0324	-0.5927	-0.5927
97	Training	8.301	7.2579	-1.0431	7.2258	-1.0752	0.0298	-1.1825	-1.2189
98	Prediction	7.538	7.1943	-0.3437	-	-	0.0287	-0.3894	-0.3894
99	Training	5.137	3.6967	-1.4403	3.6084	-1.5286	0.0578	-1.6568	-1.7584
100	Training	5.446	4.438	-1.008	4.3927	-1.0533	0.043	-1.1505	-1.2022
101	Training	8.398	7.6578	-0.7402	7.6297	-0.7683	0.0366	-0.8421	-0.8741
102	Prediction	7.658	7.64	-0.018	-	-	0.0382	-0.0205	-0.0205
103	Prediction	8.046	6.5522	-1.4938	-	-	0.0366	-1.6995	-1.6995
104	Training	7.77	7.2137	-0.5563	7.1897	-0.5803	0.0414	-0.6344	-0.6619

105	Prediction	6.721	6.9921	0.2711	-	-	0.0309	0.3075	0.3075
106	Prediction	6.432	7.3827	0.9507	-	-	0.039	1.0829	1.0829
107	Training	6.328	6.4229	0.0949	6.4255	0.0975	0.027	0.1074	0.1104
108	Prediction	6.523	6.8061	0.2831	-	-	0.0293	0.3209	0.3209
109	Training	7.745	7.3673	-0.3777	7.3513	-0.3937	0.0406	-0.4306	-0.4488
110	Training	6.409	7.2099	0.8009	7.2392	0.8302	0.0354	0.9105	0.9439
111	Prediction	0.971	2.5513	1.5803	-	-	0.0664	1.8262	1.8262
112	Prediction	5.398	4.6826	-0.7154	-	-	0.0496	-0.8194	-0.8194
113	Training	5.398	4.1857	-1.2123	4.1391	-1.2589	0.037	-1.3794	-1.4325
114	Prediction	5.301	4.0535	-1.2475	-	-	0.015	-1.4036	-1.4036
115	Training	5.301	4.5268	-0.7742	4.5062	-0.7948	0.026	-0.8759	-0.8993
116	Training	5.523	5.3856	-0.1374	5.3784	-0.1446	0.0498	-0.1574	-0.1656
117	Training	5.222	4.4073	-0.8147	4.3884	-0.8336	0.0227	-0.9202	-0.9416
118	Prediction	7.62	7.6888	0.0688	-	-	0.0492	0.0788	0.0788
119	Prediction	7.921	7.1585	-0.7625	-	-	0.0395	-0.8688	-0.8688
120	Prediction	6.745	7.616	0.871	-	-	0.0558	1.001	1.001
121	Prediction	6.602	7.3767	0.7747	-	-	0.0516	0.8883	0.8883
122	Training	8.046	7.4405	-0.6055	7.4209	-0.6251	0.0314	-0.687	-0.7092
123	Prediction	6.796	6.7377	-0.0583	-	-	0.0279	-0.0661	-0.0661
124	Prediction	8.398	7.893	-0.505	-	-	0.0408	-0.5757	-0.5757
125	Prediction	8.301	7.5529	-0.7481	-	-	0.0487	-0.8565	-0.8565
126	Training	8.301	7.3521	-0.9489	7.3101	-0.9909	0.0424	-1.0828	-1.1308
127	Training	8.097	6.9791	-1.1179	6.9428	-1.1542	0.0314	-1.2684	-1.3096
128	Prediction	7.131	6.987	-0.144	-	-	0.0634	-0.1661	-0.1661
129	Prediction	6.553	6.9232	0.3702	-	-	0.0527	0.4247	0.4247
130	Training	7.347	6.9967	-0.3503	6.9728	-0.3742	0.064	-0.4043	-0.4319
131	Training	6.824	6.8505	0.0265	6.8519	0.0279	0.0491	0.0303	0.0319
132	Prediction	7.328	6.7463	-0.5817	-	-	0.0475	-0.6655	-0.6655
133	Training	5.097	4.073	-1.024	4.0032	-1.0938	0.0638	-1.1818	-1.2624
134	Prediction	5.097	4.6585	-0.4385	-	-	0.0264	-0.4963	-0.4963

135	Training	5.046	4.6883	-0.3577	4.6709	-0.3751	0.0466	-0.409	-0.429
136	Training	3.252	3.814	0.562	3.8212	0.5692	0.0127	0.6316	0.6397
137	Prediction	5.046	4.6428	-0.4032	-	-	0.0181	-0.4544	-0.4544
138	Training	5	4.1052	-0.8948	4.0718	-0.9282	0.036	-1.0176	-1.0556
139	Prediction	5	3.9241	-1.0759	-	-	0.0538	-1.2351	-1.2351
140	Training	5	3.9923	-1.0077	3.9804	-1.0196	0.0117	-1.1319	-1.1453
141	Prediction	4.959	4.2838	-0.6752	-	-	0.0424	-0.7704	-0.7704
142	Training	4.959	4.0423	-0.9167	4.0309	-0.9281	0.0122	-1.03	-1.0427
143	Prediction	4.959	4.5182	-0.4408	-	-	0.027	-0.499	-0.499
144	Training	4.921	5.1932	0.2722	5.2044	0.2834	0.0395	0.3101	0.3228
145	Prediction	4.886	4.1765	-0.7095	-	-	0.0144	-0.7981	-0.7981
146	Prediction	4.854	3.9309	-0.9231	-	-	0.0117	-1.0368	-1.0368
147	Prediction	4.796	3.8765	-0.9195	-	-	0.0314	-1.0432	-1.0432
148	Training	4.77	3.4844	-1.2856	3.4182	-1.3518	0.049	-1.472	-1.5478
149	Prediction	3.208	1.7371	-1.4709	-	-	0.1719	-1.8049	-1.8049
150	Prediction	4.77	4.0424	-0.7276	-	-	0.0193	-0.8204	-0.8204
151	Training	4.745	3.3211	-1.4239	3.2672	-1.4778	0.0365	-1.6198	-1.6811
152	Prediction	4.745	5.2423	0.4973	-	-	0.0464	0.5686	0.5686
153	Training	4.721	3.6651	-1.0559	3.6457	-1.0753	0.018	-1.1898	-1.2116
154	Prediction	4.721	5.8083	1.0873	-	-	0.0549	1.2489	1.2489
155	Training	3.201	4.2476	1.0466	4.2614	1.0604	0.013	1.1764	1.1919
156	Training	4.721	5.3189	0.5979	5.3421	0.6211	0.0374	0.6804	0.7069
157	Prediction	4.721	3.6527	-1.0683	-	-	0.0182	-1.2039	-1.2039
158	Training	6.523	6.8123	0.2893	6.826	0.303	0.0453	0.3306	0.3463
159	Prediction	6.523	6.4472	-0.0758	-	-	0.0483	-0.0868	-0.0868
160	Training	7.046	6.803	-0.243	6.7873	-0.2587	0.0607	-0.2799	-0.298
161	Prediction	8	6.457	-1.543	-	-	0.0537	-1.7712	-1.7712
162	Training	7.222	6.6017	-0.6203	6.5642	-0.6578	0.0571	-0.7133	-0.7565
163	Prediction	7.523	6.6215	-0.9015	-	-	0.051	-1.0334	-1.0334
164	Prediction	7.097	7.4147	0.3177	-	-	0.0454	0.3632	0.3632

165	Prediction	5.745	7.7725	2.0275	-	-	0.0437	2.3152	2.3152
166	Prediction	7	6.1207	-0.8793	-	-	0.0529	-1.0089	-1.0089
167	Prediction	5.187	5.8955	0.7085	-	-	0.0378	0.8066	0.8066
168	Prediction	5.886	7.0071	1.1211	-	-	0.0425	1.2794	1.2794
169	Training	6.699	6.9115	0.2125	6.9203	0.2213	0.0399	0.2421	0.2522
170	Prediction	6.301	6.5445	0.2435	-	-	0.0444	0.2781	0.2781
171	Training	6.155	7.108	0.953	7.1455	0.9905	0.0379	1.0849	1.1276
172	Training	5.357	5.719	0.362	5.7291	0.3721	0.0271	0.4099	0.4213
173	Training	7.097	6.7812	-0.3158	6.7698	-0.3272	0.035	-0.3589	-0.372
174	Training	7.523	6.8627	-0.6603	6.8385	-0.6845	0.0353	-0.7507	-0.7782
175	Training	4.824	6.3867	1.5627	6.4333	1.6093	0.029	1.7708	1.8236
176	Training	4.854	5.8681	1.0141	5.9048	1.0508	0.0349	1.1527	1.1944
177	Prediction	4.699	3.9896	-0.7094	-	-	0.012	-0.7969	-0.7969
178	Prediction	4.678	5.4004	0.7224	-	-	0.041	0.8238	0.8238
179	Training	4.678	3.4033	-1.2747	3.3615	-1.3165	0.0317	-1.4465	-1.494
180	Prediction	4.678	3.6817	-0.9963	-	-	0.0156	-1.1213	-1.1213
181	Training	4.678	3.9275	-0.7505	3.9094	-0.7686	0.0235	-0.8481	-0.8685
182	Training	3.187	3.9072	0.7202	3.952	0.765	0.0585	0.8288	0.8803
183	Training	4.658	3.7439	-0.9141	3.7275	-0.9305	0.0176	-1.0298	-1.0483
184	Prediction	4.602	4.9825	0.3805	-	-	0.029	0.4312	0.4312
185	Training	4.538	3.9264	-0.6116	3.9183	-0.6197	0.013	-0.6874	-0.6965
186	Prediction	4.523	3.954	-0.569	-	-	0.0118	-0.6391	-0.6391
187	Prediction	4.481	3.8288	-0.6522	-	-	0.012	-0.7327	-0.7327
188	Training	4.481	4.7968	0.3158	4.8123	0.3313	0.0467	0.3612	0.3789
189	Training	4.469	4.2572	-0.2118	4.248	-0.221	0.0415	-0.2416	-0.2521
190	Training	4.444	3.5303	-0.9137	3.5108	-0.9332	0.021	-1.0311	-1.0532
191	Prediction	4.432	4.9722	0.5402	-	-	0.0305	0.6126	0.6126
192	Training	4.409	3.421	-0.988	3.3961	-1.0129	0.0246	-1.117	-1.1452
193	Prediction	4.398	3.6971	-0.7009	-	-	0.149	-0.8484	-0.8484
194	Prediction	4.387	3.9946	-0.3924	-	-	0.0138	-0.4412	-0.4412

195	Prediction	4.337	4.2478	-0.0892	-	-	0.0472	-0.102	-0.102
196	Training	4.328	4.2351	-0.0929	4.2337	-0.0943	0.0151	-0.1045	-0.1061
197	Prediction	4.328	5.0912	0.7632	-	-	0.0258	0.8635	0.8635
198	Training	4.328	3.4154	-0.9126	3.3916	-0.9364	0.0254	-1.0323	-1.0592
199	Prediction	4.292	4.4446	0.1526	-	-	0.0515	0.175	0.175
200	Training	4.292	4.0214	-0.2706	4.0179	-0.2741	0.0125	-0.3041	-0.308
201	Training	4.284	3.6168	-0.6672	3.6045	-0.6795	0.0181	-0.7519	-0.7658
202	Prediction	4.268	4.9672	0.6992	-	-	0.0283	0.7921	0.7921
203	Prediction	3.131	3.1188	-0.0122	-	-	0.0202	-0.0138	-0.0138
204	Training	4.252	4.6155	0.3635	4.6341	0.3821	0.0487	0.4161	0.4374
205	Prediction	4.244	4.2319	-0.0121	-	-	0.0157	-0.0136	-0.0136
206	Training	4.237	3.9406	-0.2964	3.9372	-0.2998	0.0113	-0.3329	-0.3367
207	Prediction	4.222	4.342	0.12	-	-	0.0509	0.1376	0.1376
208	Training	4.215	4.3592	0.1442	4.3613	0.1463	0.0145	0.1622	0.1646
209	Training	4.194	3.9754	-0.2186	3.9712	-0.2228	0.0188	-0.2464	-0.2511
210	Training	3.131	4.0764	0.9454	4.091	0.96	0.0152	1.0638	1.0802
211	Prediction	4.149	4.9356	0.7866	-	-	0.0663	0.909	0.909
212	Prediction	4.125	4.4093	0.2843	-	-	0.0184	0.3205	0.3205
213	Prediction	4.119	4.0992	-0.0198	-	-	0.012	-0.0222	-0.0222
214	Training	4.119	3.3617	-0.7573	3.3429	-0.7761	0.0242	-0.856	-0.8773
215	Prediction	5.004	5.8795	0.8755	-	-	0.032	0.9937	0.9937
216	Prediction	6.046	5.9912	-0.0548	-	-	0.0438	-0.0626	-0.0626
217	Training	6.523	6.7226	0.1996	6.7338	0.2108	0.053	0.229	0.2418
218	Prediction	5.42	6.4885	1.0685	-	-	0.0958	1.2548	1.2548
219	Training	6	6.3598	0.3598	6.3711	0.3711	0.0306	0.408	0.4209
220	Training	7.398	7.3737	-0.0243	7.3727	-0.0253	0.0404	-0.0277	-0.0288
221	Prediction	7.398	7.1874	-0.2106	-	-	0.0481	-0.2411	-0.2411
222	Prediction	6.699	6.8044	0.1054	-	-	0.0664	0.1218	0.1218
223	Training	7.398	6.8546	-0.5434	6.8223	-0.5757	0.0562	-0.6246	-0.6618
224	Training	6.745	7.3509	0.6059	7.3873	0.6423	0.0567	0.6966	0.7384

225	Training	4.114	3.8545	-0.2595	3.8498	-0.2642	0.0177	-0.2924	-0.2976
226	Prediction	4.097	4.9624	0.8654	-	-	0.0414	0.9869	0.9869
227	Training	4.092	4.6012	0.5092	4.6272	0.5352	0.0486	0.583	0.6128
228	Prediction	4.056	4.1082	0.0522	-	-	0.0299	0.0592	0.0592
229	Training	3.119	3.2242	0.1052	3.2273	0.1083	0.0281	0.1192	0.1226
230	Training	4.027	3.9039	-0.1231	3.9023	-0.1247	0.0131	-0.1383	-0.1402
231	Prediction	4.018	4.6732	0.6552	-	-	0.0182	0.7384	0.7384
232	Training	4	4.9827	0.9827	5.0084	1.0084	0.0255	1.1116	1.1407
233	Prediction	3.996	4.1796	0.1836	-	-	0.046	0.21	0.21
234	Prediction	3.959	4.2912	0.3322	-	-	0.0506	0.3807	0.3807
235	Training	3.959	4.8951	0.9361	4.9055	0.9465	0.011	1.0511	1.0628
236	Prediction	3.921	3.7327	-0.1883	-	-	0.0167	-0.2121	-0.2121
237	Training	3.921	4.3504	0.4294	4.3577	0.4367	0.0168	0.4835	0.4918
238	Training	3.873	4.7517	0.8787	4.8003	0.9273	0.0524	1.008	1.0637
239	Prediction	3.854	3.5437	-0.3103	-	-	0.0169	-0.3494	-0.3494
240	Training	3.851	3.1576	-0.6934	3.0729	-0.7781	0.1089	-0.8202	-0.9204
241	Prediction	3.842	4.5648	0.7228	-	-	0.022	0.8161	0.8161
242	Training	3.83	4.7659	0.9359	4.8129	0.9829	0.0478	1.071	1.1248
243	Training	3.824	3.5994	-0.2246	3.5964	-0.2276	0.0134	-0.2525	-0.2559
244	Training	3.114	4.5894	1.4754	4.6293	1.5153	0.0263	1.6696	1.7147
245	Prediction	3.824	2.9053	-0.9187	-	-	0.0443	-1.0493	-1.0493
246	Prediction	3.815	3.8553	0.0403	-	-	0.0141	0.0454	0.0454
247	Training	3.785	4.3697	0.5847	4.3888	0.6038	0.0316	0.6635	0.6851
248	Prediction	3.775	3.7844	0.0094	-	-	0.0166	0.0106	0.0106
249	Prediction	3.114	4.3575	1.2435	-	-	0.042	1.4186	1.4186
250	Training	3.762	3.8913	0.1293	3.8938	0.1318	0.0188	0.1457	0.1485
251	Prediction	3.759	4.1893	0.4303	-	-	0.0105	0.483	0.483
252	Prediction	3.745	3.2104	-0.5346	-	-	0.0195	-0.6029	-0.6029
253	Training	3.745	4.1348	0.3898	4.1427	0.3977	0.0199	0.4396	0.4486
254	Prediction	3.721	5.2347	1.5137	-	-	0.0316	1.7176	1.7176

255	Training	3.699	2.0269	-1.6721	1.597	-2.102	0.2045	-2.0934	-2.6316
256	Training	3.678	4.1086	0.4306	4.1293	0.4513	0.0459	0.4923	0.516
257	Prediction	3.678	3.9035	0.2255	-	-	0.0355	0.2564	0.2564
258	Prediction	3.658	3.2378	-0.4202	-	-	0.0153	-0.4728	-0.4728
259	Prediction	3.633	4.4573	0.8243	-	-	0.0125	0.9263	0.9263
260	Training	3.631	4.168	0.537	4.1824	0.5514	0.0261	0.6076	0.6239
261	Prediction	3.62	4.0308	0.4108	-	-	0.0181	0.463	0.463
262	Training	3.62	2.9334	-0.6866	2.8502	-0.7698	0.108	-0.8118	-0.9101
263	Training	3.602	4.0142	0.4122	4.0192	0.4172	0.012	0.4631	0.4687
264	Prediction	3.602	3.2243	-0.3777	-	-	0.0307	-0.4284	-0.4284
265	Prediction	3.565	2.6178	-0.9472	-	-	0.0715	-1.0977	-1.0977
266	Prediction	3.509	4.3338	0.8248	-	-	0.0221	0.9314	0.9314
267	Training	3.503	3.9452	0.4422	3.9504	0.4474	0.0117	0.4967	0.5025
268	Training	3.481	3.2936	-0.1874	3.2908	-0.1902	0.0148	-0.2108	-0.214
269	Prediction	3.481	4.6608	1.1798	-	-	0.0252	1.3343	1.3343
270	Prediction	3.092	4.1911	1.0991	-	-	0.0177	1.2384	1.2384
271	Training	3.432	4.0082	0.5762	4.0205	0.5885	0.021	0.6502	0.6642
272	Training	3.398	3.6453	0.2473	3.6505	0.2525	0.0207	0.279	0.2849
273	Prediction	3.284	4.4141	1.1301	-	-	0.0168	1.2727	1.2727
274	Training	3.261	3.0172	-0.2438	3.0118	-0.2492	0.0215	-0.2753	-0.2813
275	Training	3.071	3.7696	0.6986	3.7908	0.7198	0.0295	0.7918	0.8159
276	Prediction	3.027	3.4117	0.3847	-	-	0.0156	0.433	0.433
277	Training	3.009	3.1229	0.1139	3.126	0.117	0.0263	0.1289	0.1324
278	Prediction	6.469	3.4825	-2.9865	-	-	0.0625	-3.4443	-3.4443
279	Training	4.387	3.1668	-1.2202	3.1366	-1.2504	0.0241	-1.3793	-1.4134
280	Training	5.921	4.0239	-1.8971	3.98	-1.941	0.0226	-2.1428	-2.1924
281	Prediction	4.444	2.5941	-1.8499	-	-	0.0379	-2.106	-2.106
282	Prediction	4.194	3.0875	-1.1065	-	-	0.0183	-1.247	-1.247
283	Prediction	4.31	3.3298	-0.9802	-	-	0.0245	-1.1082	-1.1082
284	Training	3.602	3.4659	-0.1361	3.4622	-0.1398	0.0259	-0.154	-0.1581

285	Prediction	3.62	3.9583	0.3383	-	-	0.052	0.388	0.388
286	Prediction	3.194	2.8895	-0.3045	-	-	0.0509	-0.349	-0.349
287	Prediction	2.444	3.0055	0.5615	-	-	0.0539	0.6446	0.6446
288	Training	3.523	3.2212	-0.3018	3.211	-0.312	0.0329	-0.3427	-0.3543
289	Prediction	2.854	3.5693	0.7153	-	-	0.0391	0.8148	0.8148
290	Prediction	3.26	3.1289	-0.1311	-	-	0.0312	-0.1487	-0.1487
291	Training	2.699	3.8821	1.1831	3.9669	1.2679	0.0669	1.3676	1.4657
292	Training	2	2.8694	0.8694	3.0365	1.0365	0.1612	1.06	1.2637
293	Prediction	1.569	3.3507	1.7817	-	-	0.0263	2.0162	2.0162
294	Prediction	3.886	3.3507	-0.5353	-	-	0.0263	-0.6057	-0.6057
295	Prediction	2.921	2.6343	-0.2867	-	-	0.0484	-0.3282	-0.3282
296	Prediction	3.409	3.8592	0.4502	-	-	0.0675	0.5206	0.5206
297	Prediction	3.469	3.265	-0.204	-	-	0.0344	-0.2318	-0.2318
298	Training	3.553	3.4255	-0.1275	3.4216	-0.1314	0.0294	-0.1445	-0.1489
299	Training	3.638	3.4755	-0.1625	3.4703	-0.1677	0.0313	-0.1843	-0.1903
300	Training	1.886	2.9203	1.0343	2.9864	1.1004	0.06	1.1913	1.2673
301	Prediction	3.538	3.3221	-0.2159	-	-	0.0345	-0.2454	-0.2454
302	Training	2.921	3.5273	0.6063	3.5542	0.6332	0.0425	0.6918	0.7225
303	Prediction	2.602	2.4296	-0.1724	-	-	0.0422	-0.1967	-0.1967
304	Prediction	3.137	2.4296	-0.7074	-	-	0.0422	-0.8072	-0.8072
305	Prediction	1.921	3.4538	1.5328	-	-	0.0303	1.7381	1.7381
306	Prediction	2.018	3.484	1.466	-	-	0.0254	1.6583	1.6583
307	Prediction	2.337	3.484	1.147	-	-	0.0254	1.2974	1.2974
308	Training	3.721	3.2991	-0.4219	3.2845	-0.4365	0.0334	-0.4792	-0.4958
309	Training	3.886	3.2991	-0.5869	3.2788	-0.6072	0.0334	-0.6666	-0.6897

Figure S1: Different graphs associated with model 1 (a) graph of experimental vs residual values (b) Y-scrambling plot

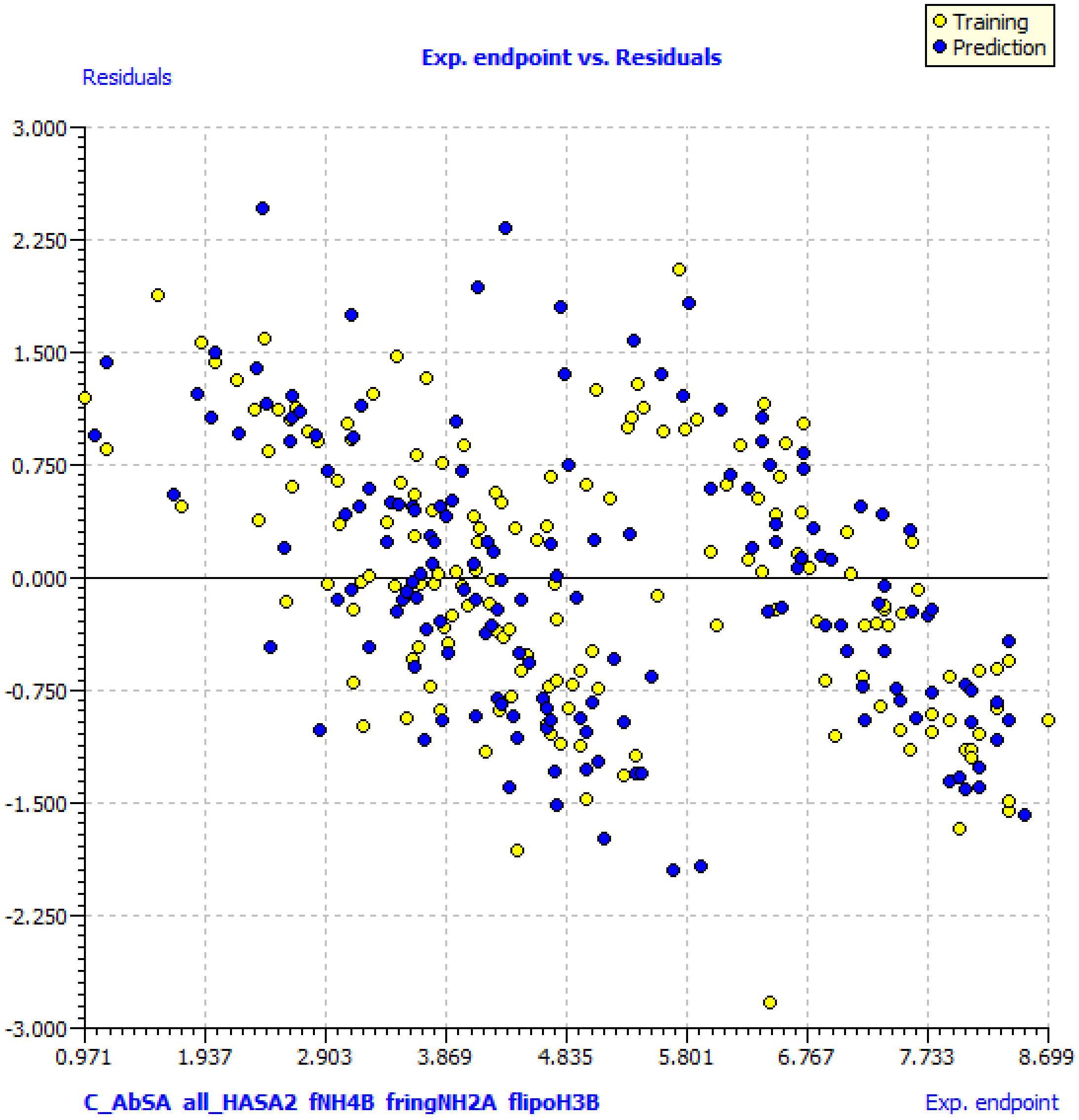

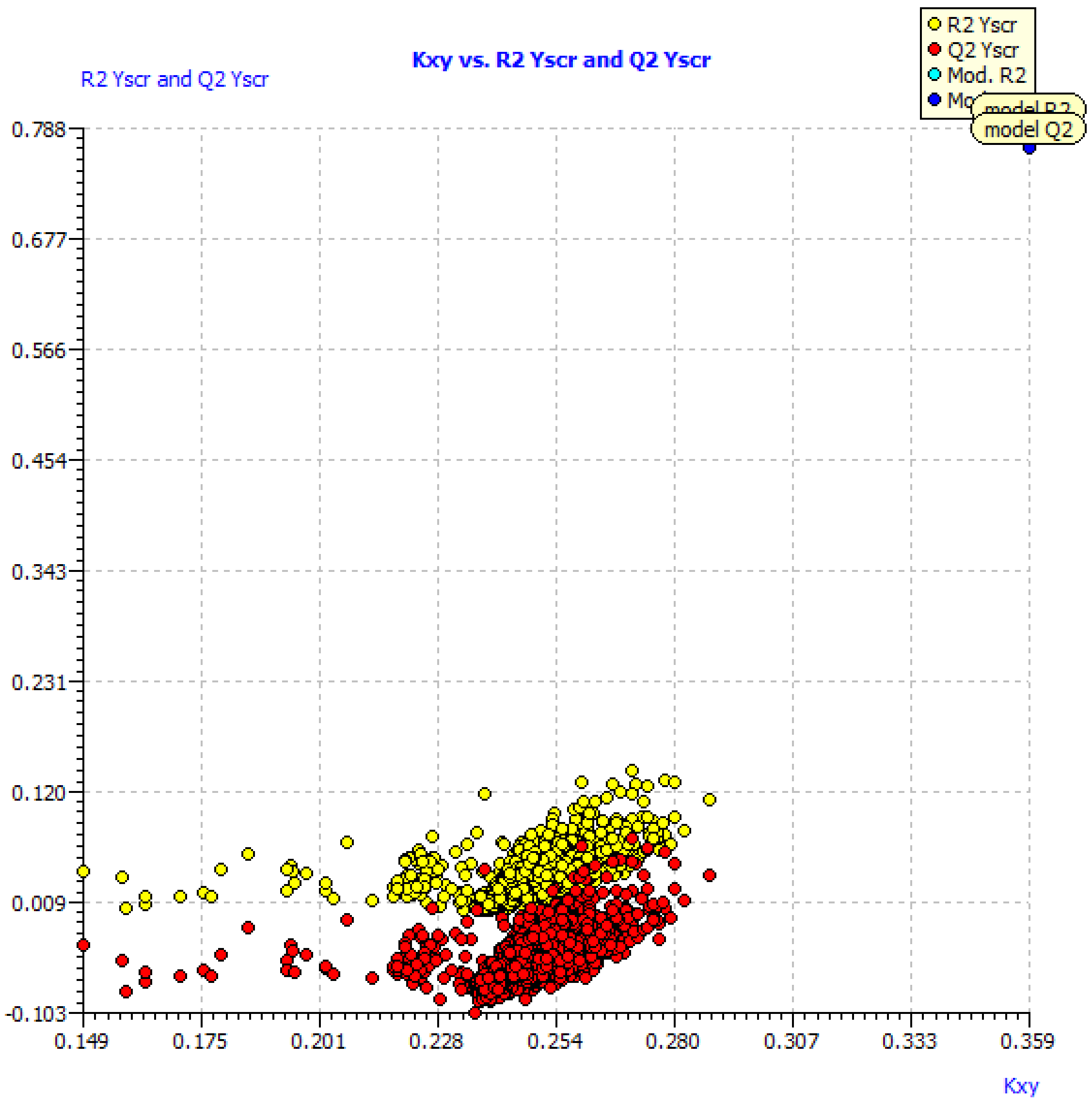
Kxy vs. R2 Yscr and Q2 Yscr
R2 Yscr and Q2 Yscr
R2 Yscr
Q2 Yscr
Mod. R2
model Q2
0.788
0.677
0.566
0.454
0.343
0.231
0.120
0.009
-0.103
0.149
0.175
0.201
0.228
0.254
0.280
0.307
0.333
0.359
Kxy

Figure S2: Different graphs associated with model 2 (a) graph of experimental vs residual values (b) Y-scrambling plot

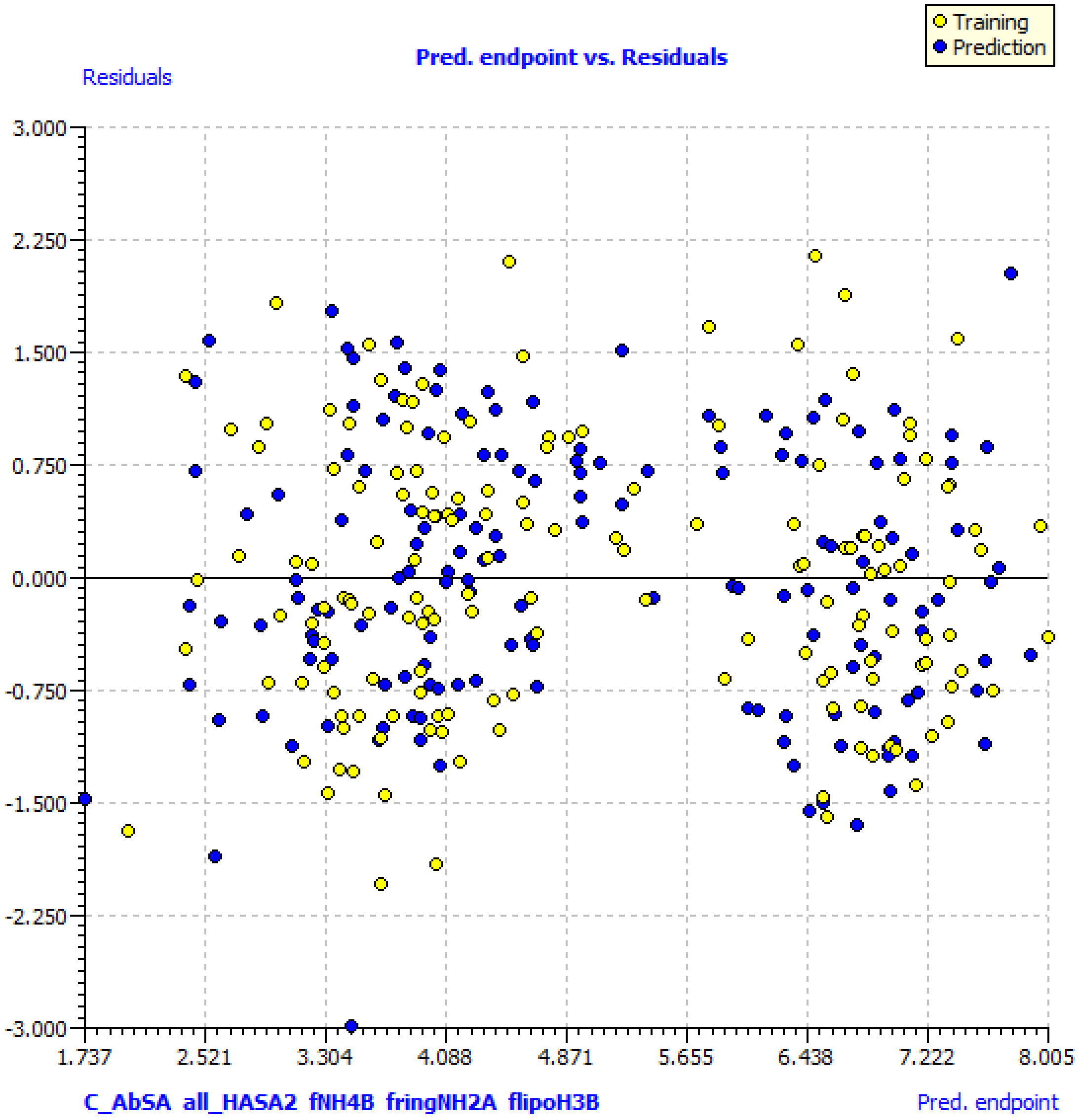

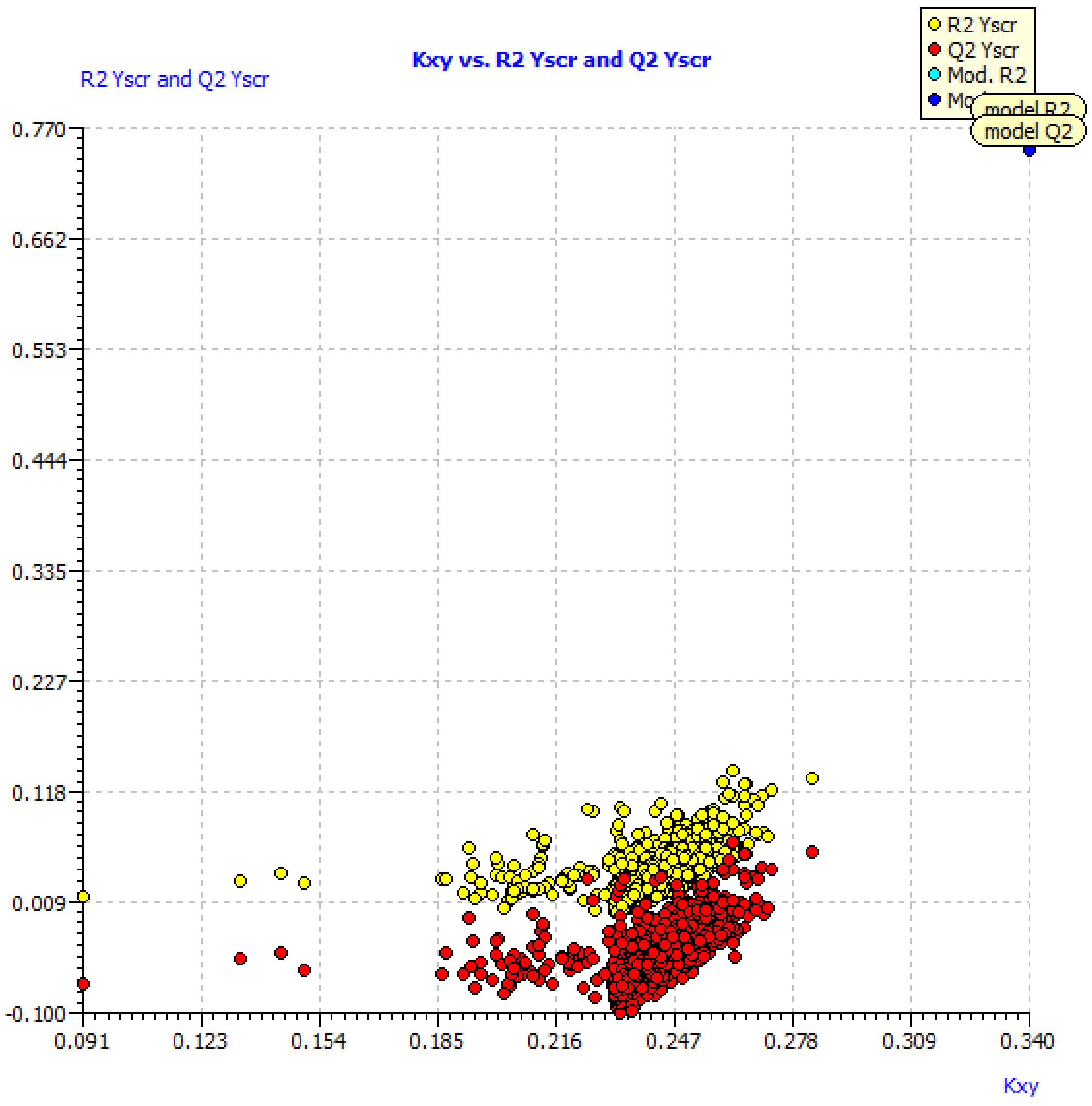

Kxy vs. R2 Yscr and Q2 Yscr
R2 Yscr and Q2 Yscr
R2 Yscr
Q2 Yscr
Mod. R2
Mo
model R2
model Q2
0.770
0.662
0.553
0.444
0.335
0.227
0.118
0.009
-0.100
0.091
0.123
0.154
0.185
0.216
0.247
0.278
0.309
0.340
Kxy

Statistical parameters for used for validation of QSAR models:

$$R^2 = 1 - \frac{\sum(y_i - \hat{y_i})^2}{\sum(y_i - \bar{y})^2}$$

where y_i are the observed values of the response, ȳ the corresponding average, ŷ are the calculated values

$$Q^2 = 1 - \frac{\sum(y_i - \hat{y_i})^2}{\sum(y_i - \bar{y})^2}$$

where y_i are the observed values of the response, ȳ the corresponding average, ŷ are the values predicted for each object when it is not in the training set.

$$Q_{F1}^2 = 1 - \frac{\sum_{i=1}^{n_{EXT}}(y_i - \hat{y_i})^2}{\sum_{i=1}^{n_{EXT}}(y_i - \bar{y}_{TR})^2}$$

where y_i are the observed values of the response, ȳ the corresponding average, ŷ are the calculated values

$$Q_{F2}^2 = 1 - \frac{\sum_{i=1}^{n_{EXT}}(y_i - \hat{y_i})^2}{\sum_{i=1}^{n_{EXT}}(y_i - \bar{y}_{EXT})^2}$$

where y_i are the observed values of the response, ȳ the corresponding average, ŷ are the calculated values

$$Q_{F3}^2 = 1 - \frac{\left[\sum_{i=1}^{n_{EXT}}(y_i - \hat{y_i})^2\right]/n_{EXT}}{\left[\sum_{i=1}^{n_{TR}}(y_i - \bar{y}_{TR})^2\right]/n_{TR}}$$

where y_i are the observed values of the response, ȳ the corresponding average, ŷ are the calculated values

$$CCC = \frac{2\sum_{i=1}^{n_{EXT}}(y_i - \bar{y})(\hat{y}_i - \bar{\hat{y}})}{\sum_{i=1}^{n_{EXT}}(y_i - \bar{y})^2 + \sum_{i=1}^{n_{EXT}}(\hat{y}_i - \bar{\hat{y}})^2 + n_{EXT}(\bar{y} - \bar{\hat{y}})^2}$$

$$k = \frac{\sum_{i=i}^{n_{EXT}} y_i \hat{y}_i}{\sum_{i=1}^{n_{EXT}} \hat{y}_i^2}$$

$$k' = \frac{\sum_{i=i}^{n_{EXT}} y_i \hat{y}_i}{\sum_{i=1}^{n_{EXT}} y_i^2}$$

$$r_m^2 = r^2\left(1 - \sqrt{r^2 - r_0^2}\right)$$

$$\overline{r_m^2} = \frac{(r_m^2 + r_m'^2)}{2}$$

$$RMSE = \sqrt{\frac{\sum_{i=1}^{n_{EXT}}(y_i - \hat{y}_i)^2}{n_{EXT}}}$$

$$MAE = \frac{\sum_{i=1}^{n_{EXT}}|y_i - \hat{y}_i|}{n_{EXT}}$$

where y_i are the observed values of the response, $\bar{y}$ the corresponding average, $\hat{y}$ are the calculated value
